# Stage‐Adaptive Janus Microneedle System for Redox‐Immune Regulation and Mitochondrial Protection in Infected Diabetic Wound Healing

**DOI:** 10.1002/advs.202600076

**Published:** 2026-07-29

**Authors:** Mengting Yin, Yu Zhang, Xinyu Qu, Jiayi Liu, Zhongyi Sun, Haibo Liu, Ziyan Chen, Jing Ru, Jingwen Han, Bingqiang Lu, Yan Lu, Yan Wang, Xinyu Zhao, Feng Chen

**Affiliations:** ^1^ Shanghai Key Laboratory of Craniomaxillofacial Development and Diseases Shanghai Stomatological Hospital & School of Stomatology Fudan University Shanghai People's Republic of China; ^2^ Center for Orthopaedic Science and Translational Medicine Department of Orthopaedics Shanghai Tenth People's Hospital School of Medicine Tongji University Shanghai People's Republic of China; ^3^ State Key Laboratory of High Performance Ceramics and Superfine Microstructure Shanghai Institute of Ceramics Chinese Academy of Science Shanghai People's Republic of China; ^4^ Shenghua Zizhu Academy Shanghai People's Republic of China

**Keywords:** infected diabetic wounds, mitochondrial bioenergetics, redox–immune regulation, single‐atom nanozymes, stage‐adaptive microneedles

## Abstract

Infected diabetic wounds are sustained by a vicious cycle of hyperglycemia‐driven bacterial infection, persistent oxidative stress, and excessive inflammation, which collectively disrupt the ordered progression of tissue repair. Here, we engineered a stage‐adaptive Janus microneedle patch (MN‐FeSAC‐PPE) to enable a staged therapeutic process from early antibacterial intervention to subsequent redox–immune microenvironment remodeling and regenerative tissue repair. This stage‐adaptive design integrates Fe single‐atom nanozymes (Fe‐SACs) into the microneedle base to rapidly kill bacteria using near‐infrared light, which activates reactive oxygen species (ROS) production, enabling rapid antibacterial activity against wound pathogens. Meanwhile, propolis extract‐loaded (PPE) tips deliver antioxidant bioactive compounds into the wound bed to mitigate oxidative stress, modulate the redox‐immune microenvironment, and support the inflammatory‐to‐regenerative transition. In vitro, MN‐FeSAC‐PPE enhanced antioxidant defense, suppressed pro‐inflammatory factors, and protected fibroblasts from oxidative stress‐induced mitochondrial dysfunction. Transcriptomic analysis further supported reduced inflammatory signaling and enhanced metabolism‐related programs. In *S. aureus*‐infected diabetic wounds, NIR‐activated MN‐FeSAC‐PPE accelerated wound closure, promoted angiogenesis and collagen remodeling, and alleviated inflammation. These findings establish a stageadaptive redox‐immune and bioenergetic regulatory microneedle platform for infected diabetic wound repair.

## Graphical abstract

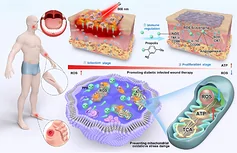

## 1 Introduction

Infected diabetic wounds are not merely bacteria‐colonized skin lesions, but are perpetuated by a vicious cycle of oxidative stress, immune response, and metabolic imbalance induced by diabetes [1, 2, 3]. Bacterial invasion triggers a rapid oxidative response to eliminate pathogens, but persistent hyperglycemia and infection exacerbate reactive oxygen species (ROS) production, inflammatory signaling, and mitochondrial damage [4, 5]. Excessive ROS impairs mitochondrial membrane potential and ATP production in repair‐related cells, weakens fibroblast migration and endothelial angiogenic activity, and sustains inflammatory activation that delays immune resolution [6], collagen remodeling, and re‐epithelialization [7, 8]. Therefore, the central challenge in infected diabetic wound healing is not simply how to kill bacteria or scavenge ROS, but how to reconcile the stage‐dependent need for bactericidal oxidation with the subsequent requirement for redox‐immune and bioenergetic restoration.

Diabetic wound healing normally proceeds through coordinated hemostatic, inflammatory, proliferative, and remodeling phases [9]. However, under hyperglycemic and infected conditions, these phases become pathologically uncoupled. During hemostasis and early inflammation, the hyperglycemic wound milieu favors bacterial growth and weakens host defenses, prolonging infection and inflammatory activation [10]. In the inflammatory‐to‐proliferative transition, excessive pro‐inflammatory mediators and ROS impair mitochondrial metabolism, reducing ATP supply for fibroblast migration, endothelial angiogenesis, granulation tissue formation, and collagen synthesis [11, 12, 13, 14]. In the remodeling phase, persistent metabolic and inflammatory abnormalities disrupt collagen maturation and delay tissue reconstruction [15, 16]. To tackle these challenges, an array of therapeutic approaches have been devised for diabetic wound management, including (i) antimicrobial dressings to control the infection [17, 18], (ii) bioactive molecules to modulate the microenvironment [19, 20, 21], (iii) functional materials to facilitate the regeneration [22, 23, 24], (iv) stem cell‐based therapy and (v) bioengineered skin substitutes [25, 26, 27]. However, current therapeutic strategies focus only on antibacterial activity, superficial closure, or single‐factor microenvironment modulation, which often fail to fully restore tissue repair. Therefore, an effective therapeutic system should therefore address the sequential pathological demands of infected diabetic wounds: early pathogen elimination, suppression of excessive inflammatory and oxidative stress, restoration of mitochondrial energy metabolism, and promotion of regenerative remodeling.

Nanozyme‐based antibacterial therapy provides an attractive route for the first step of this therapeutic sequence. Single‐atom nanozymes possess atomically dispersed catalytic centers and high enzyme‐like efficiency, enabling local catalytic reactions with improved activity compared with conventional nanomaterials [28, 29]. In particular, Fe single‐atom nanozymes (Fe‐SACs) can activate infection‐associated H_2_O_2_ to generate bactericidal ROS, while near‐infrared irradiation further enhances photothermal conversion and catalytic antibacterial efficacy [30]. This ROS‐generating capability is advantageous for rapid bacterial clearance in infected wounds [31]. However, the same oxidative chemistry that benefits antibacterial defense may become detrimental if the host wound microenvironment remains under persistent oxidative stress. Thus, Fe‐SACs‐mediated antibacterial intervention must be paired with a complementary strategy that protects host cells from ROS‐induced mitochondrial injury and inflammatory persistence after infection control.

To complement Fe‐SACs‐mediated antibacterial oxidation, a host‐protective regulatory component is required to counteract persistent oxidative stress and inflammatory activation after pathogen suppression. Natural bioactive small molecules target specific cells and molecular pathways to modulate key processes within the tissue microenvironment [32, 33]. PPE is rich in flavonoids, phenolic acids, and caffeic acid derivatives. It exhibits antioxidant, anti‐inflammatory, immunomodulatory, and bioenergetic regulatory properties. In this study, it is not presented merely as an additional bioactive compound but as a host‐protective module designed to complement early FeSACs‐mediated antibacterial oxidation [34, 35, 36, 37]. By mitigating excessive ROS and inflammatory stress following pathogen clearance, PPE helps preserve mitochondrial function and supports the activities of fibroblasts, endothelial cells, and other repair‐associated cells, thereby facilitating tissue regeneration [38, 39]. Consequently, PPE represents a promising candidate for integration into biomaterials targeting diabetic wound healing and advanced skin dressings.

Building upon this concept, we developed a stage‐adaptive Janus microneedle patch (MN‐FeSAC‐PPE), to integrate catalytic antibacterial therapy with redox–immune and bioenergetic regulation for infected diabetic wound repair. In this compartmentalized architecture, Fe‐SACs were incorporated into the microneedle base to provide NIR‐enhanced photothermal and nanozyme‐catalytic antibacterial activity, whereas PPE‐loaded microneedle tips locally delivered antioxidant bioactive compounds into the wound bed. This design was intended not as a simple combination of two functional materials, but as a phase‐matched therapeutic system that first exploits ROS for bacterial elimination and subsequently restrains excessive oxidative and inflammatory stress to restore mitochondrial bioenergetic homeostasis and support tissue regeneration. The antibacterial performance, antioxidant capacity, inflammatory regulation, mitochondrial protection, fibroblast migration, endothelial tube formation, and in vivo wound repair efficacy of MN‐FeSAC‐PPE were systematically evaluated. Furthermore, transcriptomic profiling of LPS‐stimulated RAW264.7 cells was used to interrogate whether FeSACs‐based treatment attenuates inflammatory signaling while restoring energy metabolism‐related pathways. Together, this study presents a stage‐adaptive Janus microneedle strategy that links infection control to redox‐immune remodeling and bioenergetic restoration for infected diabetic wound repair.

## 2 Results and Discussion

### 2.1 Synthesis and Characterization of Fe‐SACs

Fe‐SACs were prepared by employing a pyrolysis method for the controlled liberation of metal atoms (as depicted in Scheme 1a) [40]. Porous graphitic carbon nitride (g‐C_3_N_4_) nanosheets were first prepared from urea pyrolysis. The g‐C_3_N_4_ template was then dispersed in tris‐buffer solution, treated ultrasonically, and coated with a polydopamine (PDA) layer, forming PDA@g‐C_3_N_4_. Fe^3+^ ions were anchored onto the PDA layer, forming the Fe^3+^‐PDA@g‐C_3_N_4_ precursor. Pyrolysis of this precursor at 1000°C under nitrogen resulted in nitrogen‐doped carbon scaffolds with atomically dispersed Fe ions as active sites. The obtained Fe‐SACs feature an ultrathin, porous 2D sheet‐like morphology with wrinkled surfaces, as confirmed by transmission electron microscopy (TEM) imaging (Figure 1a–c and Figure S1). This observation is further supported by the results from aberration‐corrected scanning transmission electron microscopy (AC‐STEM), which reveal bright spots corresponding to individual Fe atoms are clearly visible throughout the carbon nanosheets, confirming the atomic dispersion of Fe in the Fe‐SACs catalyst (Figure 1d and Figure S2). Elemental mapping images reveal a uniform distribution of Fe and N species within the carbon nanosheets (Figure 1e), the effective embedding of nitrogen atoms within the carbon lattice.

**SCHEME 1:**
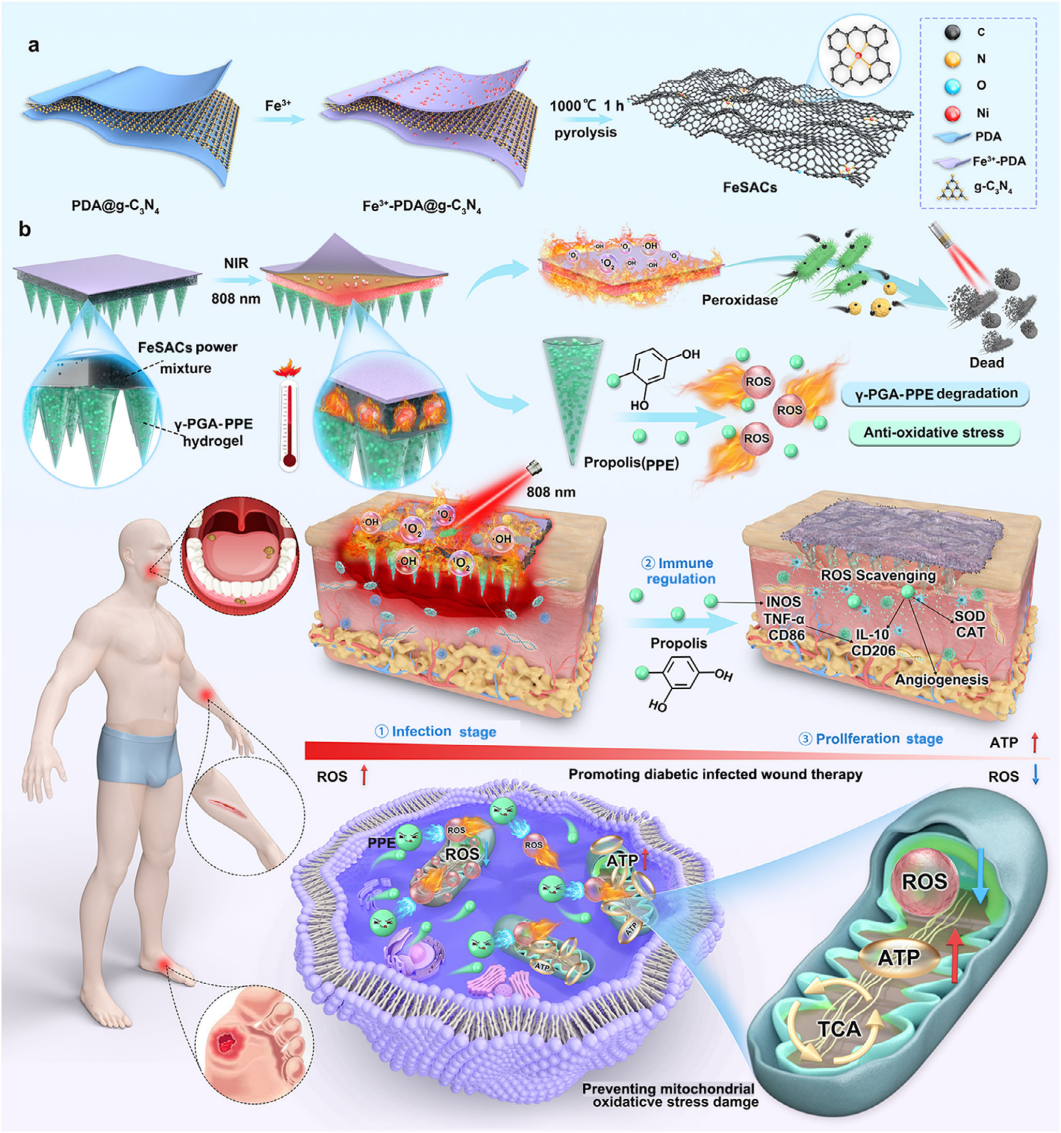
Schematic diagram of the infected diabetic wound healing process programmed by the dual‐layer multifunctional microneedle patch (MN‐FeSAC‐PPE). (a) Synthesis process of Fe‐SACs nanoenzymes. (b) Therapeutic mechanism of MN‐FeSAC‐PPE in promoting wound healing.

**FIGURE 1:**
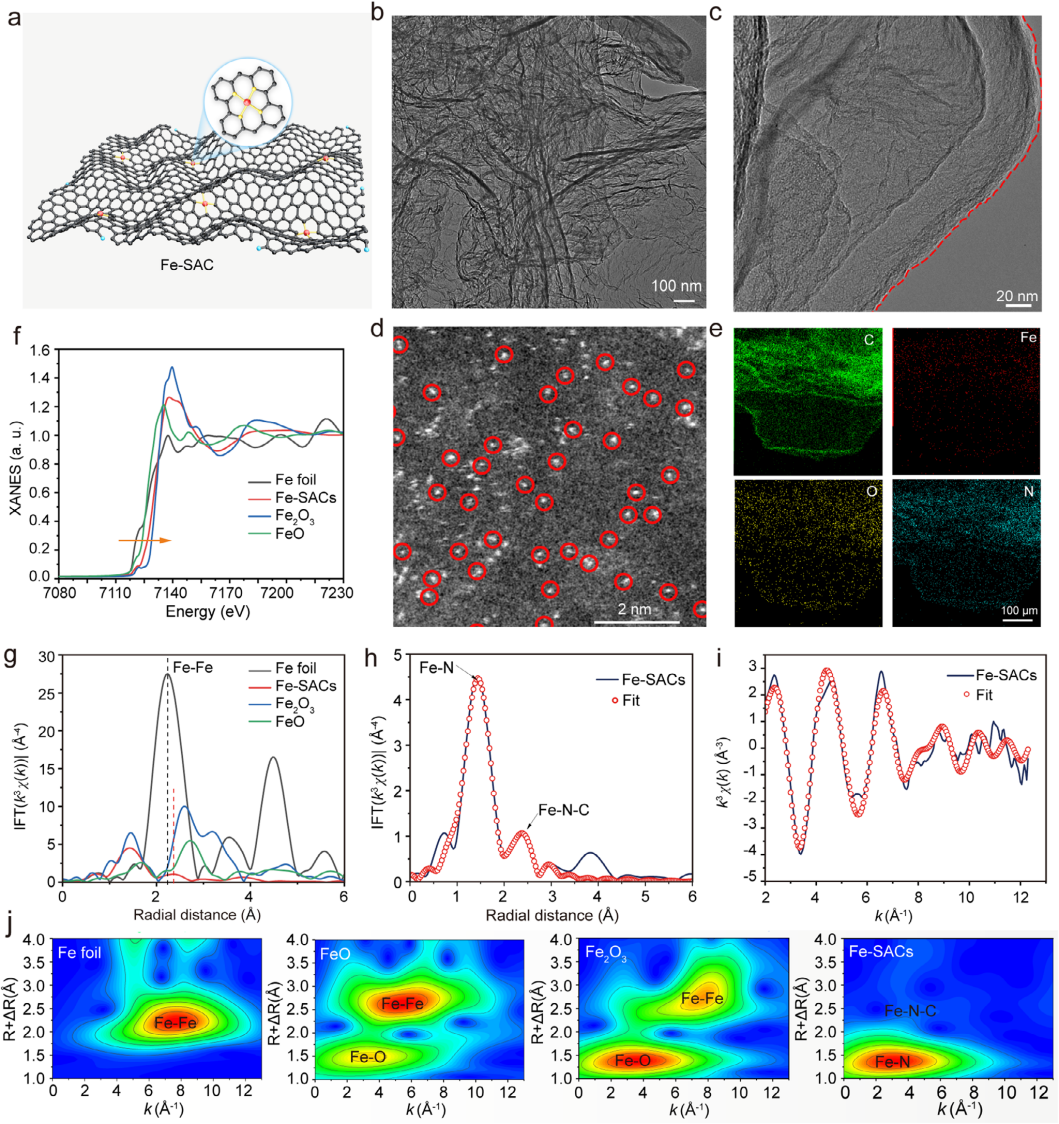
(a) Schematic diagram of the construction of Fe‐SACs. (b) TEM image of the as‐prepared Fe‐SACs. (c) Representative TEM micrograph of the as‐synthesized Fe‐SACs (scale bar: 20 nm). (d) AC‐STEM micrograph of Fe‐SACs, revealing singly dispersed iron atoms (circled in red) on the amorphous support. (e) EDS corresponding elemental mapping of synthesized Fe‐SACs. (f) Fe K‐edge XANES spectroscopy analysis of various samples (Fe‐SACs, Fe foil, FeO, Fe_2_O_3_). (g) Fourier‐transformed Fe K‐edge EXAFS spectra of Fe‐SACs. (h) EXAFS fitting result of Fe‐SACs in R space. (i) EXAFS fitting outcome of Fe‐SACs in k space. (j) Wavelet transformation Fe k‐edge EXAFS of Fe‐SACs, Fe_2_O_3_, FeO and Fe foil.

XANES analysis provided detailed information about the coordination environment and chemical state of the Fe atoms (Table S1). Figure 1f shows the XANES spectrum of Fe‐SACs compared to Fe foil, Fe_2_O_3_ and FeO references. The absorption edge of Fe‐SACs is positioned between those of standard Fe foil and Fe_2_O_3_, suggesting an iron oxidation state between +2 and +3. Figure 1g presents the Fourier transform (FT) of the k^3^‐weighted extended x‐ray absorption fine structure (EXAFS) spectra. No Fe─Fe main peaks at 2.2 and 4.5 Å are observed in the curve of Fe‐SACs, indicating that all the Fe species in the Fe‐SACs sample are present in the single‐atom state. The shift of the main peak from ∼2.8 Å in Fe_2_O_3_/FeO to ∼1.5 Å in Fe‐SACs indicates that the Fe atoms are coordinated by N atoms rather than O atoms. The fitting curve for EXAFS in R space shows that the coordination number of Fe atom is ∼4.5 in the first shell with the bonding length of ∼1.5 Å (Figure 1h). The shoulder peak at ∼2.4 Å could be corresponding to the Fe─N─C bonding in the second shell (Figure 1h). The fitting curve for EXAFS in k space shows that the strong oscillations of the Fe‐SACs sample occur at lower wavenumbers, indicating that Fe atoms are coordinated with light elements (Figure 1i). The K‐edge EXAFS spectra of the different samples further support this conclusion. The wavelet transformation plot identifies no Fe─Fe bonds in the Fe‐SACs sample, which differs markedly from Fe Foil, FeO, and Fe_2_O_3_, affirming the single‐atom dispersion of the Fe species (Figure 1j). The similarity between Fe─N bonds in the Fe‐SACs sample and Fe─O bonds in Fe_2_O_3_ sample is due to the difficulty in distinguishing nitrogen and oxygen, two adjacent elements, in EXAFS fitting.

### 2.2 NIR Irradiation Enhances Peroxidase‐Like Activity of Fe‐SACs

Fe‐SACs have attracted growing interest across diverse catalytic fields [41]. The excellent peroxidase (POD)‐like catalytic characteristics of Fe‐SACs make them potential candidates for antibacterial application [42]. The peroxidase‐mimicking performance of the as‐prepared Fe‐SACs was quantitatively examined at concentrations of 10–300 µg/mL using H_2_O_2_ as oxidant in the presence of two colorimetric substrates, 3,3',5,5′‐tetramethylbenzidine (TMB) and methylene blue (MB) (Figure 2a). Higher concentrations of Fe‐SACs significantly enhance the absorbance of oxidized TMB (oxTMB) at 652 nm, demonstrating a dose‐dependent POD‐like activity. The observed dose‐response kinetics confirm the intrinsic POD‐mimetic properties of Fe‐SACs (Figure 2b). For biomedical applications, where minimizing material exposure is critical for biosafety, an optimized lower concentration of Fe‐SACs (200 µg/mL) was selected, balancing robust catalytic activity with biocompatibility. As the concentration of H_2_O_2_ increased, the production of •OH in the system was significantly elevated, indicating that the peroxidase‐mimicking activity of Fe‐SACs is closely contingent on the concentration of H_2_O_2_ (Figure 2c). The absorbance at 664 nm for the Fe‐SACs + H_2_O_2_ + MB group was nearly flat, while the absorbance at 652 nm for the Fe‐SACs + H_2_O_2_ + TMB group was the strongest (Figure 2d and Figure S4). These results provide strong evidence that Fe‐SACs exhibit excellent POD‐like catalytic activity. The enhanced activity is attributed to the atomically dispersed Fe‐N_x_ sites, which facilitate H_2_O_2_ activation and electron transfer to TMB, analogous to natural PODs. As depicted in Figure 2e, the reaction velocity exhibits substrate concentration‐dependent kinetics, with a linear increase at low concentrations transitioning to saturation at elevated levels. Moreover, the Fe‐SACs also achieved a higher affinity (Km = 0.2177±0.3597 mm) and the maximum catalytic efficiency (V_max_ = (0.4913±0.5725) × 10^−7^ Ms^−1^), demonstrating the excellent intrinsic POD‐like activity of Fe‐SACs.

**FIGURE 2:**
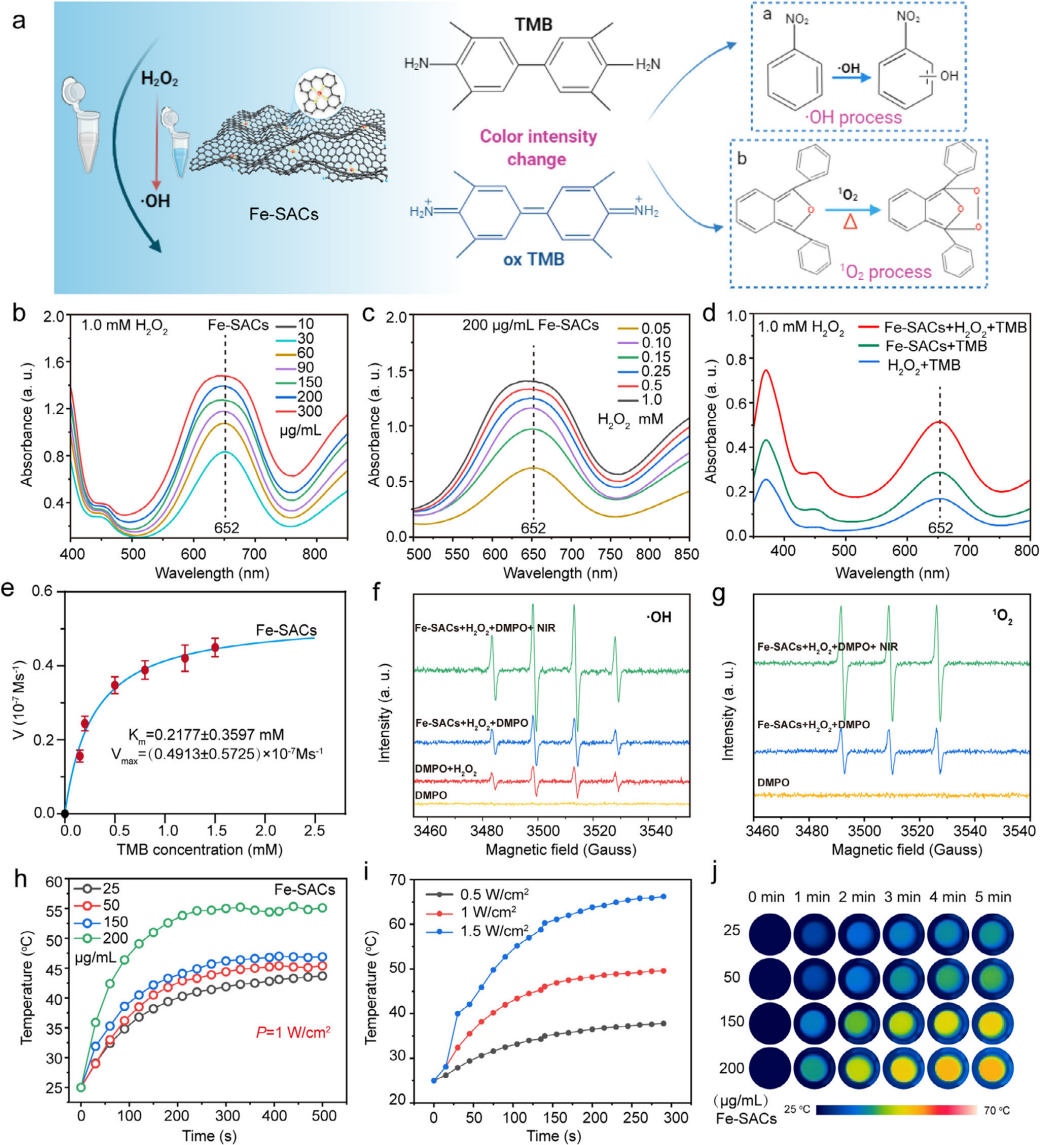
(a) A schematic diagram illustrating the peroxidase‐like catalytic activity of Fe‐SACs. (b) The POD‐like catalytic activity of Fe‐SACs at different concentrations in the presence of 1.0 mm H_2_O_2_ using TMB probes. (c) The POD‐like catalytic activity of Fe‐SACs at various concentrations of H_2_O_2_ using TMB probes. (d) UV–vis absorption spectra of TMB + H_2_O_2_, TMB + Fe‐SACs, and TMB + Fe‐SACs + H_2_O_2_. (e) TMB as substrate comparison of Km and Vmax of Fe‐SACs. (f) ESR spectrum of hydroxyl radical generated from Fe‐SACs and trapped by DMPO. (g) ESR spectrum of singlet oxygen generated from Fe‐SACs and trapped by DMPO. (h) Temperature‐elevating curves of Fe‐SACs at various concentrations under near‐infrared irradiation (1 W/cm^2^). (i) Temperature‐elevating curves of Fe‐SACs under various near‐infrared irradiation power densities (0.5, 1, and 1.5 W/cm^2^). (j) Thermal imaging of Fe‐SACs at various concentrations under near‐infrared irradiation at 1 W/cm^2^.

Subsequently, the presence of **·**OH was verified via electron spin resonance (ESR) spectroscopy using 5,5‐dimethyl‐1‐pyrroline N‐oxide (DMPO) as a ROS scavenger (Figure 2f–g). The addition of Fe‐SACs and a physiological amount of H_2_O_2_ facilitates efficient absorption of the emitted harmful **·**OH and ^1^O_2_ radicals by DMPO. The characteristic peaks of **·**OH and ^1^O_2_ were significantly enhanced upon the addition of Fe‐SACs + H_2_O_2_. Furthermore, when the Fe‐SACs + H_2_O_2_ group was subjected to photothermal stimulation, the catalyst exhibited marked specificity in generating **·**OH and ^1^O_2_ radicals, which further enhanced the POD‐like performance of Fe‐SACs upon exposure to an 808 nm near‐infrared (NIR) laser at a power density of 1.0 W/cm^2^. These results collectively confirm that Fe‐SACs nanozymes have the potential to function as catalysts with POD‐like activity, mediating H_2_O_2_ decomposition to produce ROS with strong oxidative properties. High photothermal conversion efficiency is a fundamental requirement for effective photothermal materials. As illustrated in Figure 2h–j, Fe‐SACs exhibit significant photothermal effects, with the temperature increase of the material positively correlating with its concentration. These findings suggest that Fe‐SACs are promising photothermal agents. The photothermal effects of the Fe‐SACs are attributed to the high NIR absorption capacity of the N‐doped carbon substrate, which facilitates efficient photothermal energy conversion. To evaluate their photothermal properties, the temperature rise of Fe‐SACs at various concentrations was measured under 808 nm laser irradiation (1.0 W/cm^2^, 5 min). Infrared thermography and corresponding heating profiles demonstrated that the photothermal efficiency of Fe‐SACs is dependent on both concentration and laser power density (Figure 2h–j). The Fe‐SACs suspension (200 µg/mL) exhibited a rapid temperature increase, reaching approximately 50°C upon exposure to NIR light (808 nm, 1.0 W/cm^2^) for about 4 min. This temperature rise is sufficient to achieve both photothermal antimicrobial and controlled thermal stimulation effects.

### 2.3 Synthesis and Characterization of MN‐FeSAC‐PPE Patch

A two‐step method was employed to fabricate MN‐FeSAC‐PPE hydrogel microneedle patches with a 10 × 10 array configuration [43], as illustrated in Figure 3a. The PDMS mold for the MN patch was designed with a circular overall shape. The demolded MN‐FeSAC‐PPE patch was generally square in shape, and the tips of the needles were formed as tetrahedral pyramids shape with a height of 600 µm and the spacing of 500 µm (Figure 3b, c). The pyramidal geometry facilitates rapid, precise, and minimally invasive delivery of the MN patch to the dermal layers of the skin. As shown in Figure 3d, the mechanical performance of the MN patches was evaluated with a universal testing machine. The results reveal that the pure MN patch exhibited a mechanical strength of 103.4 N, whereas the mechanical strength of the MN‐FeSAC‐PPE microneedles reached 189.1 N (1.891 N/needle). This value exceeds the minimum force required for stratum corneum penetration (0.045 N), confirming the MN‐FeSAC‐PPE microneedles possess adequate mechanical strength for effective transdermal and subcutaneous delivery [44]. The incorporation of Fe‐SACs enhanced the mechanical properties of the MN‐FeSAC‐PPE patch. This can be attributed to the chelation of iron atoms and various oxygen‐containing functional groups (e.g. carboxyl and hydroxyl groups) in the γ‐PGA matrix. The PPE complexed with FITC fluorescent protein was loaded onto the tip of the microneedle, and the successful incorporation of PPE into the tip area was clearly observed under fluorescence microscopy, with no PPE detected on the back layer of the microneedle (Figure S5). The transdermal capabilities of the MN‐FeSAC‐PPE patches were further evaluated on mouse skin. As indicated by the fluorescence images in Figure 3e, the tips loaded with Rhodamine B dye penetrated entirely into the mouse skin. H&E also confirmed that the MN‐FeSAC‐PPE patch was capable of penetrating the mouse skin surface and effectively reaching the dermal layer. To further validate the effective release of PPE from the microneedle patch tips, the microneedles loaded with Rhodamine B dye were inserted in agarose hydrogel and maintained in place for 5 min, simulating the process of skin puncture. The release dynamics at the needle tips were analyzed using laser confocal microscopy to generate 3D images (Figure 3f). As illustrated from various angles in Figure 3g, the red fluorescent tips exhibit clear diffusion and penetration downwards into the agarose hydrogel.

**FIGURE 3:**
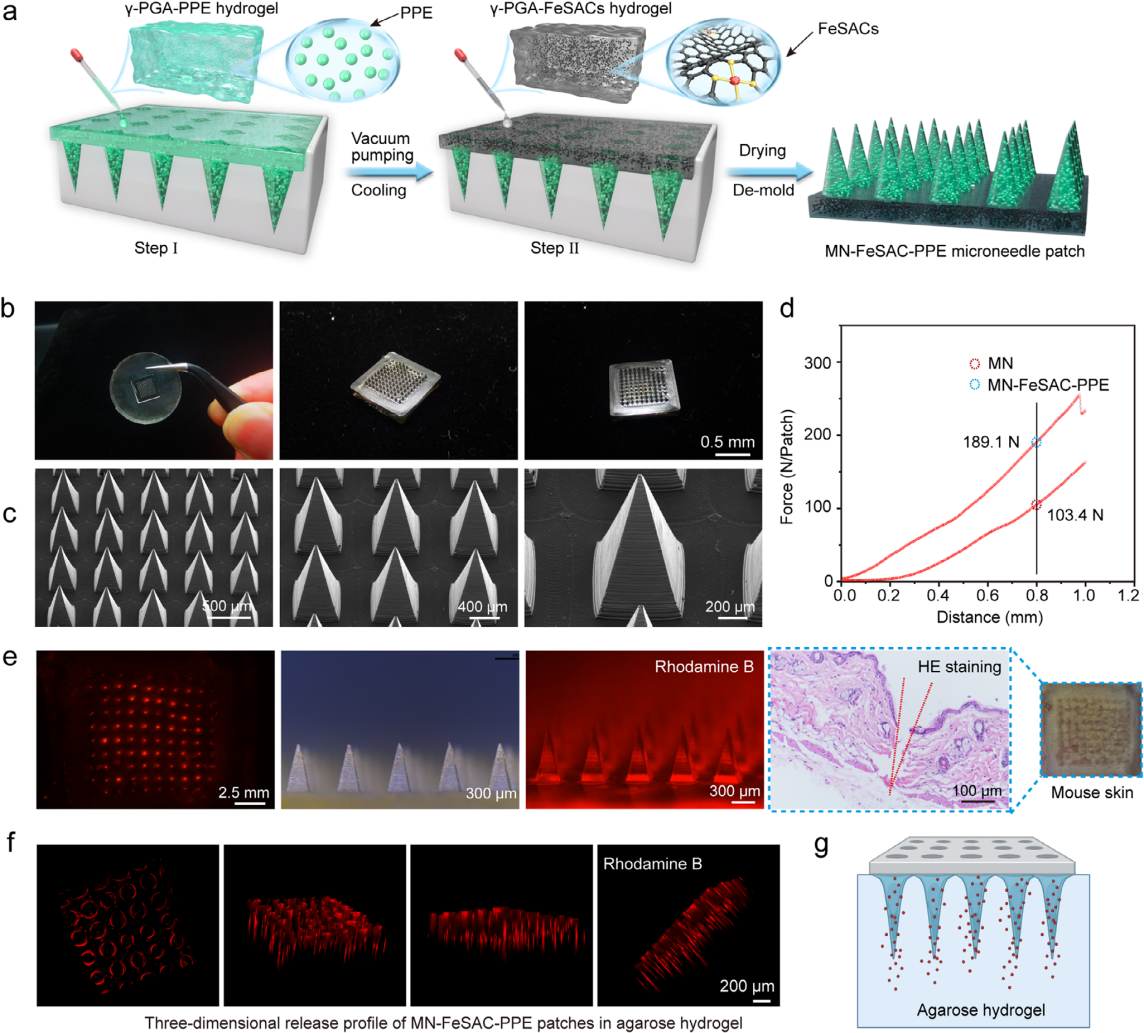
(a) Schematic illustration depicting the synthetic route to MN‐FeSAC‐PPE. (b) Optical image of the PDMS MN mold showing its microstructure. And top‐view and isometric images of the MN‐FeSAC‐PPE. (c) SEM images of MN‐FeSAC‐PPE patch. (d) Comparative force‐displacement curves of the MN‐FeSAC‐PPE and MN patches. (e) Representative bright‐field and fluorescence‐field microscopic view of mouse skin after MN‐FeSAC‐PPE insertion. H&E‐staining images of skin penetration by MN‐FeSAC‐PPE (Scale bar: 100 µm). (f) The sustained release effect of the microneedle tip in agarose hydrogel was recorded using a laser confocal 3D microscope. (g) Schematic depiction of the drug delivery behavior of the MN patch on agarose hydrogel.

### 2.4 In Vitro Photothermal Properties and Antibacterial Activities of MN‐FeSAC‐PPE

The photothermal performance of the MN‐FeSAC‐PPE patch was further evaluated by monitoring its temperature changes under NIR radiation. The temperature of the MN‐FeSAC‐PPE patch can be increased to 50°C and 64°C under NIR irradiation at the power density of 0.7 and 1 W/cm^2^ (Figure 4a). Excessive temperatures during thermal stimulation may damage tissues, leading to cellular injury or death and potentially exacerbating inflammatory responses, thereby diminishing repair efficiency. Therefore, maintaining appropriate temperatures is crucial to ensure the positive effects of heat stimulation on tissue regeneration. Consequently, a low power intensity of 0.7 W/cm^2^ was selected to ensure the sufficient yet controlled photothermal effects of MN‐FeSAC‐PPE patch (Figure 4b, c), and its photothermal conversion efficiency reached 88.6%. Thermographic imaging and photothermal curves show that the MN‐FeSAC‐PPE patch, when exposed to NIR at 0.7 W/cm^2^, achieves quicker heating and higher maximum temperatures compared to the MN and MN‐PPE patches, due to the superior photothermal effects of Fe‐SACs (Figure 4d,e).

**FIGURE 4:**
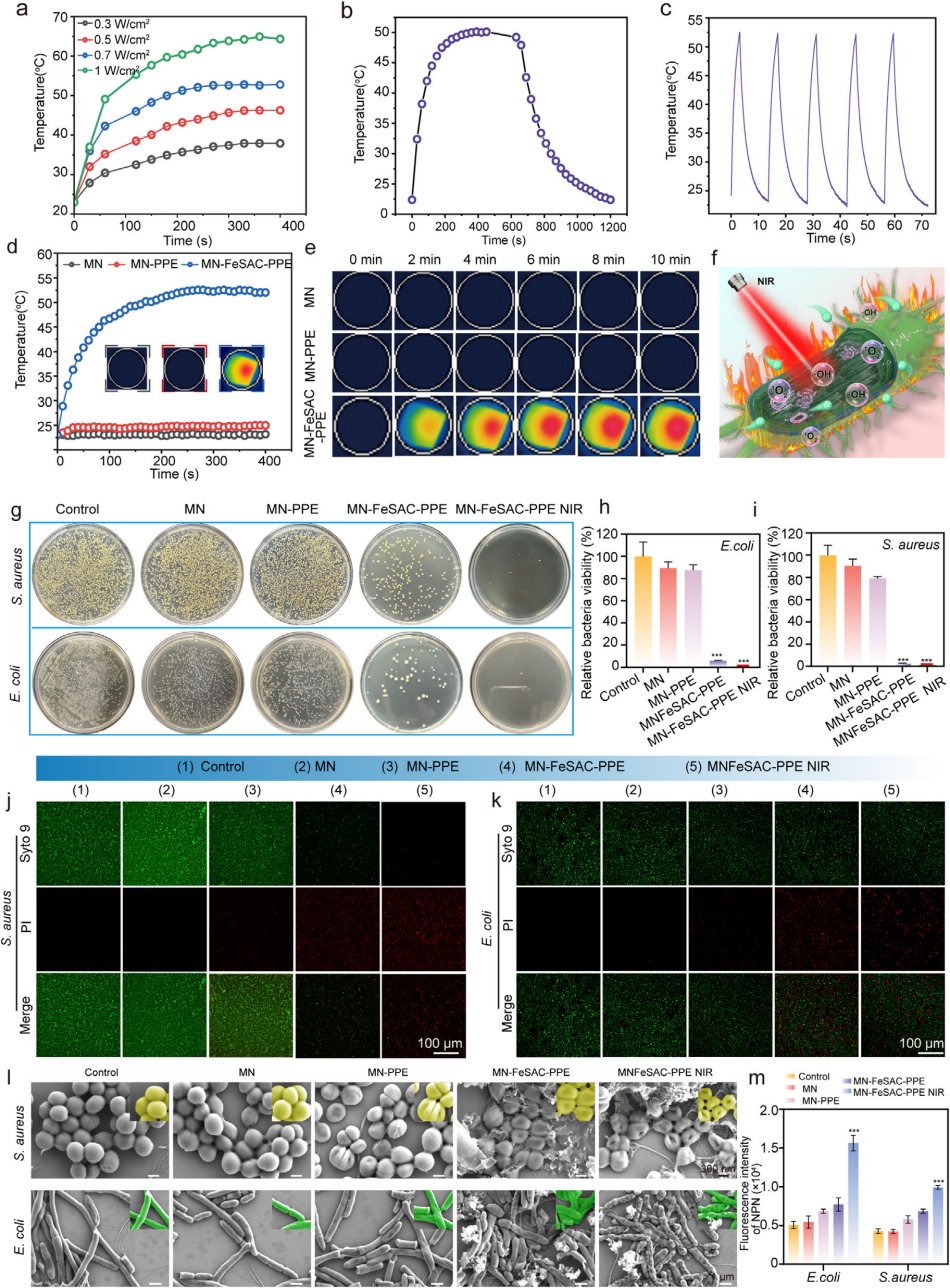
(a) photothermal temperature rise curves under 808 nm NIR with varying power densities (0.3, 0.5, 0.7, and 1 W/cm^2^). (b) The single‐cycle photothermal temperature rise curve of MN‐FeSAC‐PPE patch under NIR (808 nm, 0.7 W/cm^2^). (c) Cyclic photothermal heating/cooling curves of the MN‐FeSAC‐PPE patch under NIR. (d, e) Photothermal heating curves and infrared thermographic maps of MN, MN‐PPE, and MN‐FeSAC‐PPE patch under NIR. (f) Diagram of the proposed antibacterial mechanism of the MN‐FeSAC‐PPE patch. (g) Photographs of bacterial colonies of *S. aureus* and *E. coli* treated with different microneedle groups. (h, i) The relative viability of bacteria treated with different groups. (j, k) Fluorescence images of bacterial viability and mortality after co‐incubation of different treatment groups with *S. aureus and E. coli*. Syto 9: live bacteria; PI: dead bacteria. (l) SEM images of *S. aureus and E. coli* after treatment with different groups. (m) Influences of different microneedle patches of bacterial membrane permeability. n = 3, ^***^ *p* < 0.0001.

The aforementioned results demonstrate that the MN‐FeSAC‐PPE patch exhibits exceptional photothermal properties, which play a vital role in its antibacterial performance. Under NIR irradiation, the patch efficiently converts light energy into heat, causing a rapid increase in local temperature sufficient to kill bacteria. Moderate thermal stimulation not only disrupts bacterial cell membranes, proteins, and nucleic acids, leading to bacterial death, but also enhances the production of free radicals by Fe‐SACs nanozymes within the microneedle patch (Figure 2f,g). Therefore, the photothermal effect synergistically amplifies the nanozyme‐mediated production of free radicals, resulting in an enhanced antibacterial outcome. The results from the dilution plating method demonstrate that both the MN‐FeSAC‐PPE and the MN‐FeSAC‐PPE NIR groups exhibit excellent antibacterial effects (Figure 4g). Statistical analysis shows that the relative bacterial viability of *S. aureus* is 5.67% in the MN‐FeSAC‐PPE group, dropping dramatically to 0.07% in the MN‐FeSAC‐PPE NIR group (Figure 4h). In contrast, the relative bacterial viability in the Control group, MN group, and MN‐PPE group remains high at 100%, 89.33%, and 87.67%, respectively (Figure 4i). These results indicate that the photothermal effect enhances the nanozyme‐catalyzed production of free radicals, significantly improving the antibacterial efficacy of the MN‐FeSAC‐PPE NIR group. Additionally, the antibacterial effects of the various treatment groups were determined based on bacterial viability staining (live/dead). In the MN‐FeSAC‐PPE patch treatment groups, especially under NIR irradiation (MN‐FeSAC‐PPE NIR group), there was a significant increase in red fluorescence (dead bacteria) and a decrease in green fluorescence (live bacteria), indicating effective bacterial killing. This outcome not only supports the quantitative data obtained from the dilution plating method but also provides visual evidence of the antibacterial mechanism (Figure 4j,k). The high antibacterial efficacy of the MN‐FeSAC‐PPE patch is beneficial for effectively controlling bacteria in the early stages of wound repair. Additionally, the morphological changes of bacteria were characterized by SEM after different treatments. SEM images show that bacteria treated with MN‐FeSAC‐PPE, particularly under NIR irradiation, experienced severe membrane disruption and structural damage compared to other groups (Figure 4l). To further explore the underlying mechanism, bacterial membrane permeability was assessed, and it was confirmed that the photothermal effect enhanced membrane permeability. (Figure 4m). The photothermal treatment increases bacterial membrane permeability, which may facilitate the interaction between Fe‐SAC‐mediated catalytic ROS and bacterial cells, thereby contributing to enhanced antibacterial activity.

### 2.5 Biocompatibility, Cell Migration, and Angiogenic Ring Formation Facilitated by MN‐FeSAC‐PPE Patch

The biocompatibility of the MN, MN‐PPE, MN‐FeSAC‐PPE, and MN‐FeSAC‐PPE NIR microneedle patches were evaluated using a live/dead cell staining assay. The fluorescence microscopy images of cells treated with the different patches show green fluorescence intensities comparable to those observed in the control group (Figure 5a). Subsequently, The toxicity of the different treatment groups was quantitatively determined using the CCK‐8 assay (Figure 5b). Cell viability across all groups exceeded 90%, demonstrating that the formulated MN, MN‐PPE, and MN‐FeSAC‐PPE patches exhibit minimal cytotoxicity. Additionally, assessment of blood compatibility was conducted via an in vitro hemolysis assay across the groups. Visual inspection of the erythrocyte tubes and statistical analysis of the hemolysis rates confirmed that all patches maintained excellent blood compatibility with hemolysis rates well below the critical threshold (Figure 5c). These findings collectively indicate that the MN‐FeSAC‐PPE patches possess high biocompatibility, making them suitable for safe application in wound healing without inducing cytotoxic responses.

**FIGURE 5:**
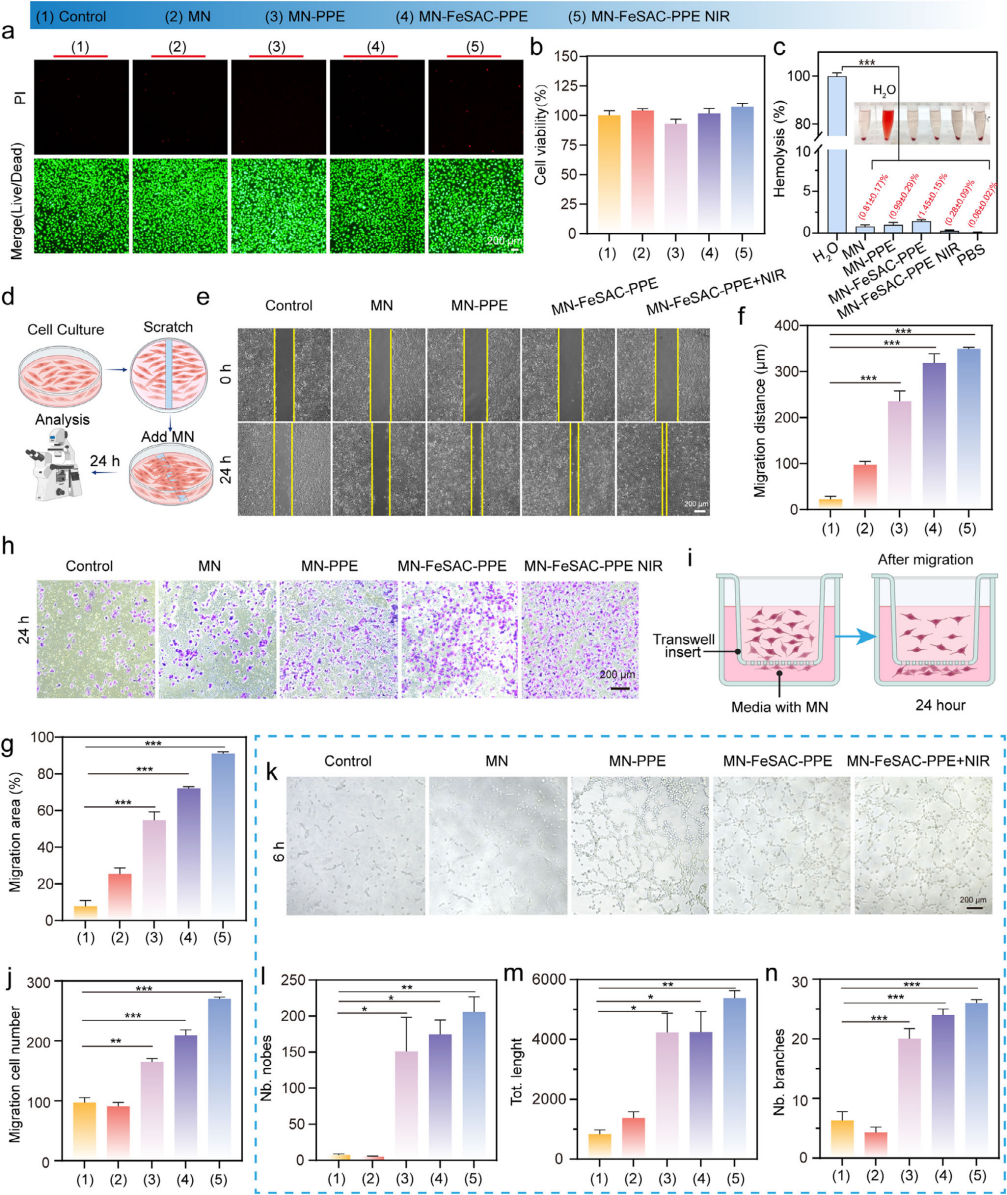
(a, b) Evaluation of in vitro cytotoxicity and biocompatibility of MN, MN‐PPE, MN‐FeSAC‐PPE, MN‐FeSAC‐PPE NIR. (c) Hemolysis of mouse blood treated with different microneedle patches. (d) Scratch assay of NIH‐3T3 cells cultured with different microneedle patches. (e‐g) Quantitative analyses of migration distance and migration area in the scratch assay. (h–j) The migratory abilities of NIH‐3T3 cells treated with different microneedle patches were further confirmed by transwell assays. (k) Tube formation images of various treatment groups. (l–n) Statistical analysis of angiogenic capacity measured by the tube formation assay. n = 3, ^*^ *p* < 0.05, ^**^ *p* < 0.01, ^***^ *p* < 0.001.

Furthermore, the migratory capacity of fibroblasts in vitro was evaluated using a scratch wound assay following treatment with the microneedle patches (Figure 5d,e). As shown in Figure 5f,g, the migration distance and migration area in the MN‐FeSAC‐PPE group and MN‐FeSAC‐PPE NIR group markedly elevated compared to the MN group and the control group. What's more, the transwell assay revealed a concordant trend with the findings from the scratch assay, with both the MN‐FeSAC‐PPE group and the MN‐FeSAC‐PPE NIR group demonstrating enhanced cell migration (Figure 5h–j). Additionally, the ability of the microneedle patches to promote angiogenesis in HUVECs was assessed (Figure 5k). In the MN‐FeSAC‐PPE NIR group, the number of nodes, the number of branches and the total tube length was 28‐fold, 4 and 6.5‐fold of that of the Control group. These findings demonstrate a markedly enhanced angiogenic capacity of the MN‐FeSAC‐PPE patch (Figure 5l–n).

### 2.6 Free Radical Scavenging Ability and Anti‐inflammatory Effects of MN‐FeSAC‐PPE Patch

Flavonoids and phenolic acids can counteract oxidative stress by neutralizing free radicals, thereby reducing inflammatory response and treating mitochondrial dysfunction [45]. The ability to scavenge free radicals of PPE solution was evaluated using a series of standard antioxidant assays including ABTS, DPPH, and PTIO as radical sources (Figure 6a). A robust free‐radical‐scavenging capability of the PPE was observed in both the DPPH and ABTS assays (Figure 6b,c). The free radical scavenging activity of the PPE solution was found to be concentration‐dependent. At a concentration of 200 µg/mL, the PPE solution exhibited a scavenging efficiency of 49.3% against ABTS radicals and 81% against DPPH radicals (Figure 6e,f). Although DPPH and ABTS assays are extensively employed to evaluate antioxidant capacity, they do not fully encompass the complete free radical scavenging abilities. Consequently, the oxygen‐centered radical PTIO was employed in subsequent evaluations. The PPE exhibited good radical scavenging activity with a scavenging efficiency of 31.33% towards PTIO at the 200 µg/mL concentration (Figure 6g). These findings collectively highlight the potent antioxidant capability of PPE, suggesting its effectiveness in neutralizing diverse free radicals, therefore potentially mitigating oxidative stress‐induced mitochondrial dysfunction.

**FIGURE 6:**
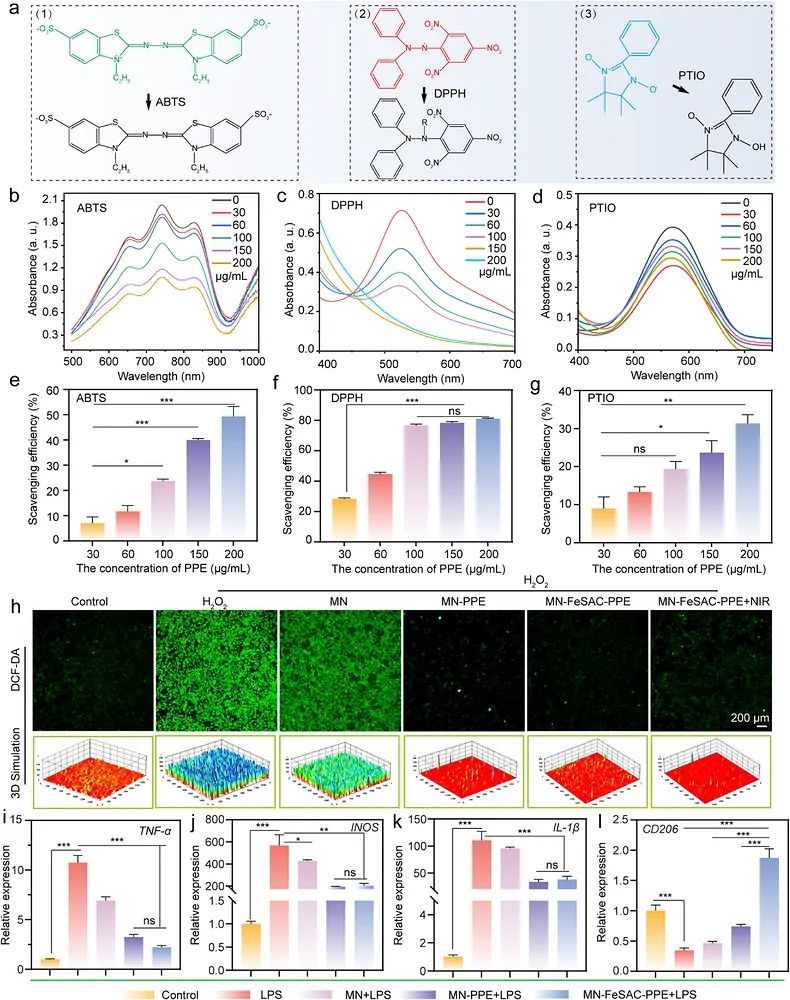
(a) The mechanism of PPE at different concentrations to scavenge three free radicals (ABTS, DPPH and PTIO). (b–g) The free radical scavenging activity of PPE at various concentrations was evaluated using UV–vis spectroscopy and quantitatively assessed for three common radicals: (b, e) DPPH, (c, f) ABTS, and (d, g) PTIO. (h) Representative ROS staining images and 3D surface plot images of NIH‐3T3 cells under different microneedle patches treatment with H_2_O_2_. ROS was stained by DCF‐DA. Scale bar: 200 µm. (i–l) The relative expression of inflammatory and macrophage polarization‐related genes/markers TNF‐α (i), INOS (j), IL‐1β (k) and CD206 (l) in macrophages under different treatment conditions. All data are shown as the mean ± standard deviation (SD). n = 3, ^*^ *p* < 0.05, ^**^ *p* < 0.01, ^***^ *p* < 0.001.

The therapeutic efficacy of the synthesized MN‐FeSAC‐PPE in protecting fibroblasts from oxidative stress‐induced mitochondrial dysfunction was evaluated by establishing a pathological oxidative microenvironment using 100 µm H_2_O_2_ treatment, either before or after administration of different microneedle patches. Subsequently, intracellular ROS levels were assessed using the ROS‐specific fluorescent probe 2′,7′‐dichlorodihydrofluorescein diacetate (DCF‐DA). The staining of DCF‐DA demonstrated that MN‐FeSAC‐PPE markedly reduced ROS levels and effectively mitigated oxidative stress (Figure 6h). Based on the ROS quantification results, the regulatory effects of MN‐FeSAC‐PPE on inflammatory responses were further investigated. Reverse‐transcription quantitative polymerase chain reaction (RT‐qPCR) was employed to measure the expression levels of key pro‐inflammatory cytokines and anti‐inflammatory mediators, thereby providing deeper insights into the role of MN‐FeSAC‐PPE in modulating inflammation under conditions of oxidative stress. RT‐qPCR analysis results confirm that stimulating macrophages with lipopolysaccharide (LPS) successfully established a pathological inflammatory microenvironment, as evidenced by the significant upregulation of pro‐inflammatory cytokines (i.e. TNF‐α, iNOS, and IL‐1β). As depicted in Figure 6i–k, treatment with MN‐PPE and MN‐FeSAC‐PPE patches significantly downregulated the expression of pro‐inflammatory markers TNF‐α, iNOS, and IL‐1β in macrophages. In contrast, the levels of anti‐inflammatory markers of CD206 and IL‐10 were markedly increased following the application of the MN‐FeSAC‐PPE patches (Figure 6l). These results indicate that the MN‐FeSAC‐PPE patch effectively attenuates the pro‐inflammatory response while enhancing anti‐inflammatory signaling in macrophages under inflammatory conditions. Flow cytometry analysis further validated these findings. As shown in Figure S6, LPS stimulation markedly increased the proportion of CD86‐positive M1 macrophages (9.42% vs. 0.41% in control), confirming successful polarization. Notably, MN‐FeSAC‐PPE treatment significantly reduced CD86‐positive cells to 5.36%, whereas MN alone showed no appreciable effect (9.47%). These results are consistent with our PCR data and collectively demonstrate that PPE effectively attenuates macrophage M1 polarization.

### 2.7 MN‐FeSAC‐PPE Attenuates Oxidative Stress‐Induced Mitochondrial Dysfunction

Under hyperglycemic conditions, excessive ROS production disrupts mitochondrial function, leading to oxidative damage and impaired cellular homeostasis. In our design, PPE is expected to scavenge excessive ROS generated by photothermal‐enhanced nanozyme antibacterial therapy and an imbalanced redox microenvironment, thereby reducing oxidative damage to cells and tissues, and restoring energy metabolism (Figure 7a). To evaluate this protective effect under pathological oxidative stress, fibroblasts were treated with 100 µm H_2_O_2_ following MN‐FeSAC‐PPE administration (Figure 7b, c). JC‐1 staining was employed to evaluate mitochondrial membrane potential (ΔΨm), a crucial indicator of mitochondrial function. In healthy cells, JC‐1 forms red aggregates, whereas in damaged or dysfunctional mitochondria, it remains green as monomers. The results demonstrate that fibroblasts treated with MN‐PPE and MN‐FeSAC‐PPE microneedle patches in a H_2_O_2_‐induced pathological oxidative microenvironment exhibited a significant increase in JC‐1 red fluorescence and a corresponding decrease in JC‐1 green fluorescence, indicating that the mitochondrial membrane potential was effectively maintained (Figure 7g, h). Consistently, MitoTracker staining revealed that these treatments alleviated H_2_O_2_‐induced mitochondrial fragmentation and restored a more intact and homogeneous mitochondrial network with stronger red fluorescence (Figure 7c). Together, these findings suggest that PPE‐containing microneedle patches protect mitochondria from oxidative stress by preserving both mitochondrial function and structural integrity.

**FIGURE 7:**
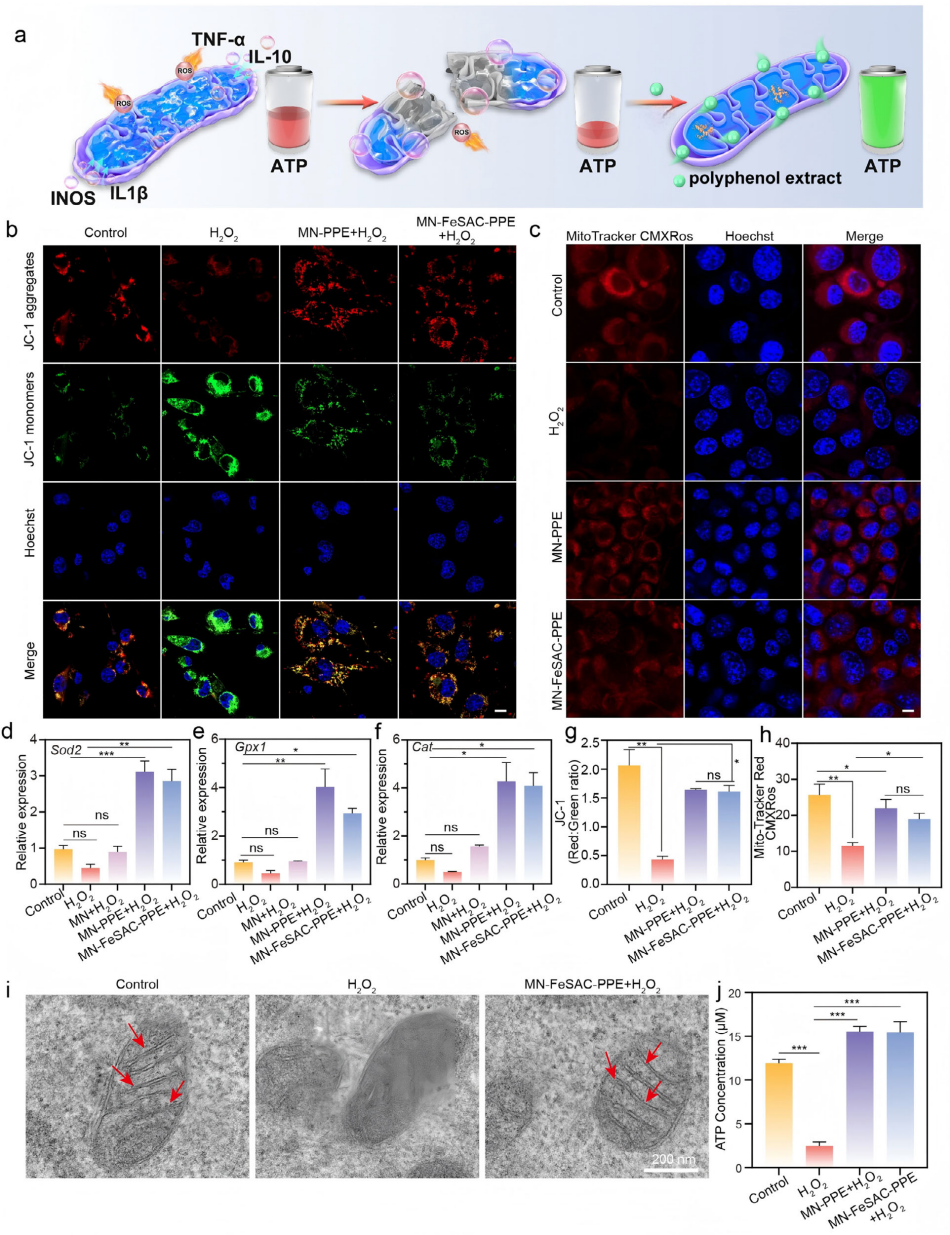
(a) Schematic illustration of the mechanism by which MN‐FeSAC‐PPE restores mitochondrial dysfunction. (b) Representative fluorescent images of NIH‐3T3 cells after various treatments analyzed by mitochondrial membrane potential assay kit with JC‐1. Scale bar: 20 µm. (c) Representative fluorescent images of NIH‐3T3 cells after various treatments analyzed by mitochondria stained with Mito‐Tracker Red. Scale bar: 20 µm. (d–f) The relative expression of antioxidant genes Sod2 (d), Gpx1 (e), and Cat (f) in NIH‐3T3 cells under different treatment conditions. (g, h) Fluorescence intensities of JC‐1 monomers and JC‐1 aggregates. (i) Representative TEM images of mitochondria of NIH‐3T3 cells. The red arrow indicates the mitochondrial cristae. Scale bar: 200 µm. (j) ATP content in NIH‐3T3 cells under different treatment conditions by measured using the ATP Luciferase Assay Kit. All data are shown as the mean ± standard deviation (SD). n = 3, ^*^ *P* < 0.05, ^**^ *P* < 0.01, ^***^ *P* < 0.001.

To further investigate the protective mechanism of MN‐FeSAC‐PPE against mitochondrial oxidative stress, the expression of antioxidant genes in fibroblasts was analyzed. Protective roles against mitochondrial oxidative stress are fulfilled by antioxidant enzymes (e.g. Sod2, Gpx1, and Cat), which collaborate to maintain the cell's redox balance. Sod2 eliminates superoxide radicals by converting them into hydrogen peroxide which is further reduced by Gpx1 to water or degraded by Cat to prevent its accumulation. These enzyme catalyzed reactions not only preserve the structure and function of mitochondria but also regulate cell survival and apoptosis, effectively safeguarding cells from oxidative stress‐induced damage. RT‐qPCR showed that MN‐FeSAC‐PPE significantly upregulated Sod2, Gpx1, and Cat, suggesting an enhanced cellular antioxidant defense (Figure 7d–f). Consistently, ATP quantification demonstrated that both MN‐FeSAC‐PPE and MN‐PPE increased ATP levels in H_2_O_2_‐treated fibroblasts compared with the model group (Figure 7j). Furthermore, the TEM images revealed that mitochondria in the MN‐FeSAC‐PPE treated group retained intact structures with well‐preserved cristae, which is significantly different from the damaged structure observed in mitochondria from the H_2_O_2_‐treated model (Figure 7i). Collectively, these findings indicate that MN‐FeSAC‐PPE alleviates oxidative stress‐induced mitochondrial damage by enhancing antioxidant capacity and preserving mitochondrial integrity and bioenergetic function.

### 2.8 Analysis of Therapeutic Efficacy of MN‐FeSAC‐PPE Using RNA Sequencing

To further validate the protective effect of MN‐FeSAC‐PPE against excessive ROS‐induced mitochondrial dysfunction and to identify potential genes involved in inflammation regulation, transcriptomic analysis was performed on RAW 264.7 cells treated with either LPS or MN‐FeSAC‐PPE under inflammatory conditions (RNA‐Seq, Figure 8). Principal component analysis (PCA) showed a clear separation between the LPS and MN‐FeSAC‐PPE groups, indicating that MN‐FeSAC‐PPE treatment induced a distinct transcriptional profile compared with LPS‐induced pro‐inflammatory activation (Figure 8b). Consistently, differential expression analysis identified a total of 1,651 differentially expressed genes (DEGs), including 905 upregulated genes and 746 downregulated genes in the MN‐FeSAC‐PPE group relative to the LPS group (Figure 8a). To further clarify the biological significance of these transcriptional changes, volcano plot and clustering heatmap analyses were performed (Figure 8c, d). The results revealed an inverse expression pattern between inflammation‐related mediators and genes associated with mitochondrial bioenergetics. In the LPS group, macrophages exhibited markedly elevated expression of pro‐inflammatory and chemotaxis‐related genes, including Tnf, Il1b, Il6, Cxcl2, and Cxcl10, along with NF‐κB‐associated regulators such as Nfkbia, Tnfrsf1b, and Tnfaip3. In contrast, these genes were substantially downregulated in the MN‐FeSAC‐PPE group, suggesting that MN‐FeSAC‐PPE effectively attenuated the LPS‐induced pro‐inflammatory transcriptional program. Notably, genes involved in antioxidant defense and mitochondrial energy metabolism, including Gpx1, Prdx1, Prdx3, Atp5mc1, Atp5mc2, Atp5if1, Ndufa4, and Idh2, were upregulated following MN‐FeSAC‐PPE treatment. These findings indicate that MN‐FeSAC‐PPE not only suppresses inflammatory activation but also supports mitochondrial redox homeostasis and ATP‐generating metabolism (Figure 8d). In line with the gene‐level results, KEGG pathway enrichment analysis further showed that the DEGs were mainly associated with inflammatory signaling and cellular metabolic regulation (Figure 8e). Several inflammation‐related pathways, including cytokine‐cytokine receptor interaction, NF‐κB signaling, TNF signaling, and IL‐17 signaling, were enriched, which was consistent with the reduced expression of representative pro‐inflammatory genes such as Tnf, Il1b, Il6, and Cxcl2 in the MN‐FeSAC‐PPE group. These results suggest that MN‐FeSAC‐PPE treatment attenuates LPS‐induced inflammatory activation at the pathway level, rather than merely reducing the expression of individual cytokines. In Figure S7, GO enrichment analysis revealed prominent enrichment of immune system process, response to stimulus, and biological regulation within the biological process category, alongside catalytic activity and molecular function regulator activity in the molecular function category. These results provide supportive transcriptomic evidence that inflammation regulation and mitochondrial metabolism‐related pathways may participate in the therapeutic effects of MN‐FeSAC‐PPE. Although metabolic improvement may be partly secondary to reduced inflammatory stress, the GO enrichment patterns suggest that coordinated regulation of immune responses and mitochondrial function‐associated processes contributes to the repair‐favorable effects of MN‐FeSAC‐PPE.

**FIGURE 8:**
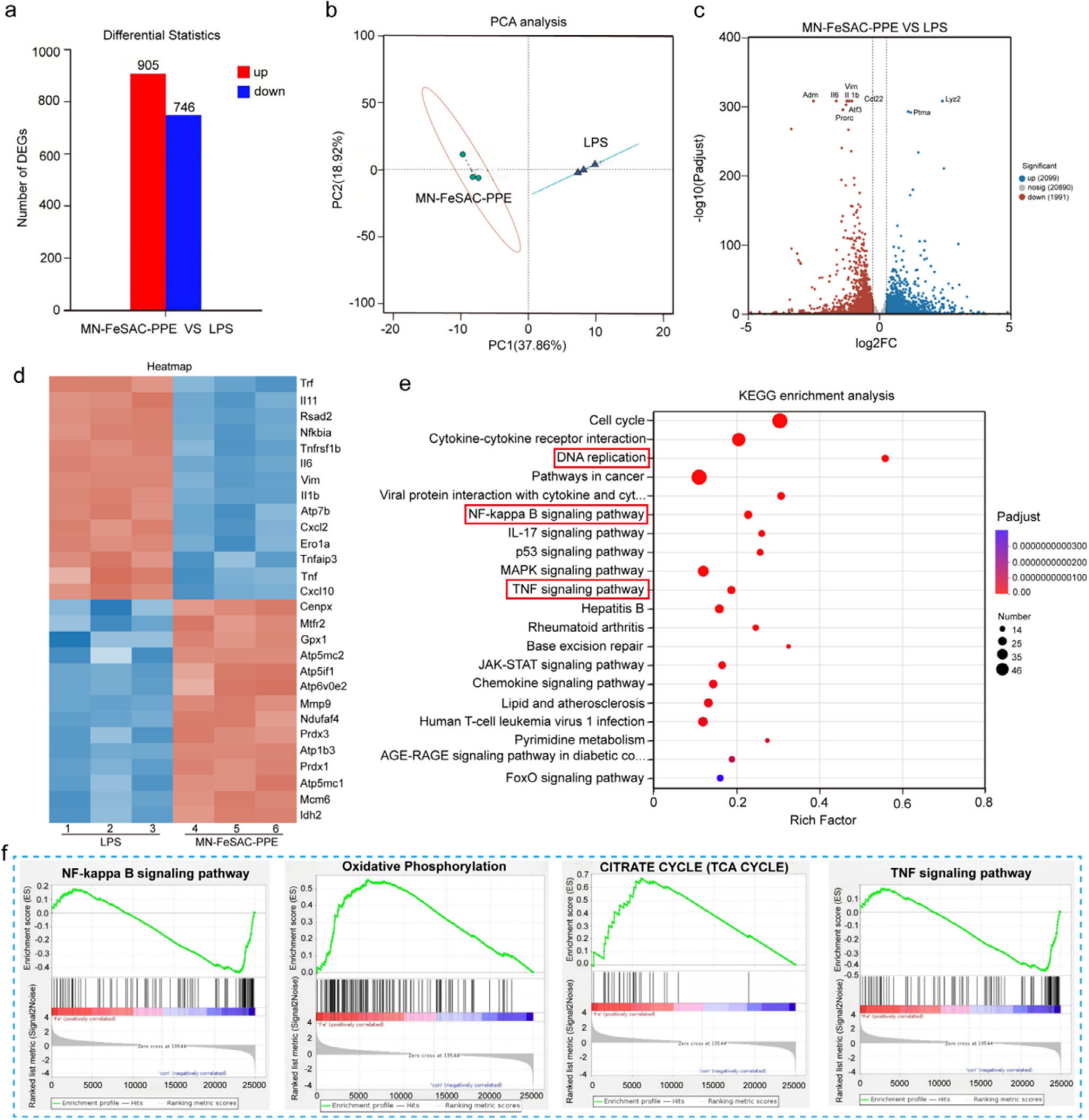
(a) The number of upregulated and downregulated genes. (b) Principal component analysis (PCA) suggesting the variance between the MN‐FeSAC‐PPE and LPS treatment groups. (c) Volcano plot showing differentially expressed genes of the MN‐FeSAC‐PPE and LPS groups. (d) The heatmap showing genes associated with inflammation and mitochondrial energy metabolism for the MN‐FeSAC‐PPE and LPS groups (n = 3). (e) Analysis of the differential gene enrichment within the KEGG pathway. (f) Gene set enrichment analysis of anti‐inflammatory and mitochondrial function‐related signaling pathways.

Moreover, gene set enrichment analysis (GSEA) provided additional evidence for the regulatory effect of MN‐FeSAC‐PPE on mitochondrial function. Oxidative phosphorylation and citrate cycle‐related gene sets were positively enriched in the MN‐FeSAC‐PPE group, indicating a potential restoration of mitochondrial bioenergetic metabolism (Figure 8f). This observation is consistent with the previous functional results showing that MN‐FeSAC‐PPE reduced intracellular ROS accumulation, preserved mitochondrial membrane potential, and increased ATP production under oxidative stress. Taken together, these transcriptomic results suggest that MN‐FeSAC‐PPE remodels the inflammatory microenvironment by suppressing cytokine‐mediated inflammatory activation while promoting mitochondrial energy metabolism, thereby facilitating the transition from an inflammatory stress state toward a repair‐favorable cellular state.

### 2.9 Healing Impact of MN‐FeSAC‐PPE Patch on Infected Diabetic Wounds

To further investigate the clinical applicability of the MN‐FeSAC‐PPE patch, we developed a full‐thickness wound model infected with *S. aureus* in insulin‐deficient diabetic mice induced by Streptozotocin (STZ). One week after STZ administration, C57BL/6J mice with fasting blood glucose levels of ≥14 mm was designated as diabetic mice. Diabetic mice infected with *S. aureus* were randomly assigned to receive microneedle‐based transdermal therapy. The in vivo drug administration and therapeutic procedures are illustrated in Figure 9a. Images of the wound areas were captured at designated time points (Figure 9b), and the corresponding wound measurements were documented (Figure 9c). Between Day 3 and 14, both the MN‐FeSAC‐PPE NIR and the MN‐FeSAC‐PPE groups exhibited superior wound healing outcomes compared to the MN and control groups. By Day 14, both the MN‐FeSAC‐PPE NIR and MN‐FeSAC‐PPE groups have demonstrated significantly enhanced wound healing. Additionally, these two groups exhibited a slightly higher healing rate than that of the MN‐PPE group. On Day 12, the MN‐FeSAC‐PPE NIR group exhibited a smallest residual wound area of merely 8.14 mm^2^. In contrast, the wound areas of the MN and control groups were approximately 51.9 mm^2^ (Figure 9d), highlighting the superior efficacy of the MN‐FeSAC‐PPE NIR treatment. Figure 9e provides a visual representation of wound area recovery, effectively illustrating the varying healing efficacies among the different treatment groups. Notably, the MN‐FeSAC‐PPE NIR group demonstrated a significantly higher wound healing rate and more accelerated healing progression compared to the MN‐FeSAC‐PPE group on each day. This could be due to the in vivo antimicrobial efficacy of the MN‐FeSAC‐PPE NIR group, which is proved by the colony sampling tests conducted on Day 3. The results indicated that the MN‐FeSAC‐PPE NIR group achieved an antimicrobial rate of 90.34%, demonstrating excellent antimicrobial performance (Figures S8 and S9). On the 14th day of treatment, wound skin specimens were harvested for comprehensive histological evaluation, which included hematoxylin‐eosin (H&E) staining and Masson's trichrome staining. As shown in Figure S11, the MN‐FeSAC‐PPE‐NIR group exhibited the smallest wound gap width and the highest relative collagen content among all groups, with statistically significant differences compared to the control and other treatment groups. After a 14‐day treatment with MN‐FeSAC‐PPE NIR, the initially immature granulation tissue developed into densely structured mature skin tissue. These results exhibited reduced wound length, increased granulation tissue thickness, and lengthened regenerated epidermis, decreased neutrophils infiltration, and enhanced collagen deposition with organized alignment. Collectively, the MN‐FeSAC‐PPE can effectively eradicate *S. aureus* infections during early treatment, subsequently alleviating oxidative stress‐induced inflammation and accelerating healing of bacterial‐infected wounds.

**FIGURE 9:**
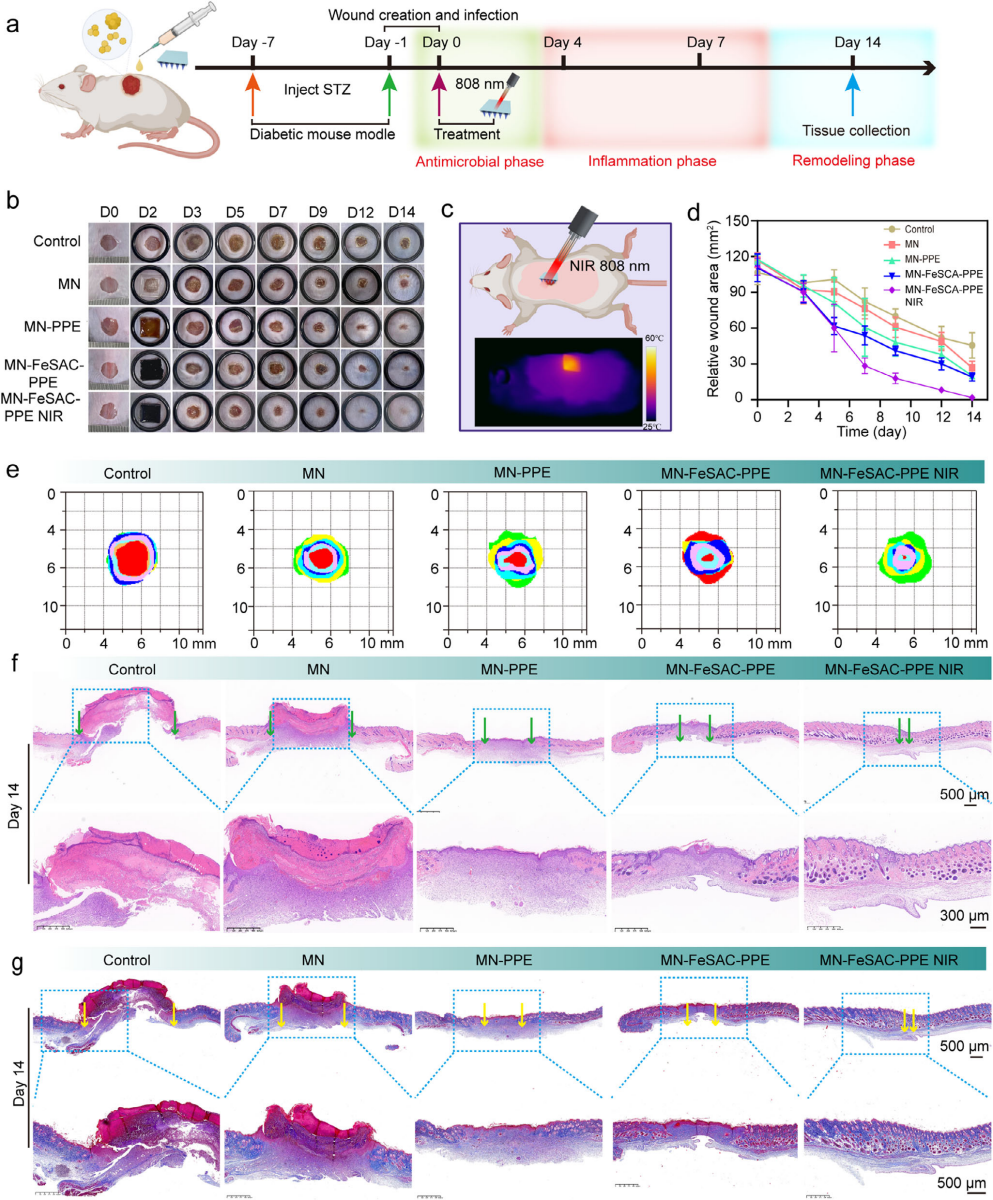
(a) The process of in vivo model establishment and the treatment protocol using different patches administration. (b) Representative images of infected diabetic wounds in different treatment groups. (c) Thermographic images of photothermal therapy using MN‐FeSAC‐PPE patch. (d) Wound area of mouse in different treatment groups. (e) A visual representation of wound area recovery in different treatment groups. (f) Representative H&E staining and Masson staining images of wounds in different treatment groups on Day 14.

Under hyperglycemic and infectious conditions, mitochondrial oxidative stress‐induced damage may impair macrophage polarization, obstructing the transition from pro‐inflammatory M1 macrophages to anti‐inflammatory M2 macrophages and thereby causing immune dysregulation. Consequently, a pivotal switch in macrophage phenotype from M1 to M2 underpins the re‐establishment of immune homeostasis in the early stages of wound repair. Therefore, the inflammatory response was evaluated by analyzing the expression of tumor necrosis factor‐α (TNF‐α) and cyclooxygenase‐2 (COX‐2) in diabetic infected wounds. The MN‐FeSAC‐PPE NIR group exhibited reduced TNF‐α and COX‐2 expression compared to the MN‐FeSAC‐PPE and MN‐PPE groups, suggesting that the infected diabetic wounds in the MN‐FeSAC‐PPE NIR group experienced a shorter inflammatory phase (Figure 10a,c,d). As mentioned before, RT‐PCR analysis revealed downregulated iNOS gene expression with concurrent upregulation of CD206 and IL‐10, indicative of M2 macrophage polarization. These results collectively underscore the anti‐inflammatory properties of PPE in MN‐FeSAC‐PPE patch. We further investigated the role of the MN‐FeSAC‐PPE patch in regulating immune homeostasis during wound healing in vivo. Immunofluorescence staining results demonstrated that the MN‐FeSAC‐PPE NIR group exhibited maximum upregulation of CD206 expression among all groups (33.47±6.24%, Figure 10b), indicating the alleviated inflammation in the MN‐FeSAC‐PPE NIR group. Intriguingly, animal blood glucose data (Figure S10) reveals a progressive decline in fasting blood glucose levels specifically in the PPE‐loaded MN patch treatment group, whereas other treatment groups maintain levels comparable to the blank control. Together with its antioxidant and anti‐inflammatory activities, the slight decrease in fasting blood glucose observed in the PPE‐containing groups may provide an additional benefit for diabetic wound repair, although the underlying mechanism requires further investigation. Additionally, CD31 and α‐SMA staining were used to scrutinize neovascularization. The results demonstrated elevated angiogenesis in the MN‐FeSAC‐PPE and MN‐FeSAC‐PPE NIR groups, which was nearly 3‐fold higher than that of the Control group (Figure 10b,f,g). Collectively, these findings demonstrate that MN‐FeSAC‐PPE patches accelerate wound healing by restoring immune homeostasis, suppressing pro‐inflammatory cytokine expression, recruiting anti‐inflammatory macrophages, and stimulating angiogenesis across all healing phases.

**FIGURE 10:**
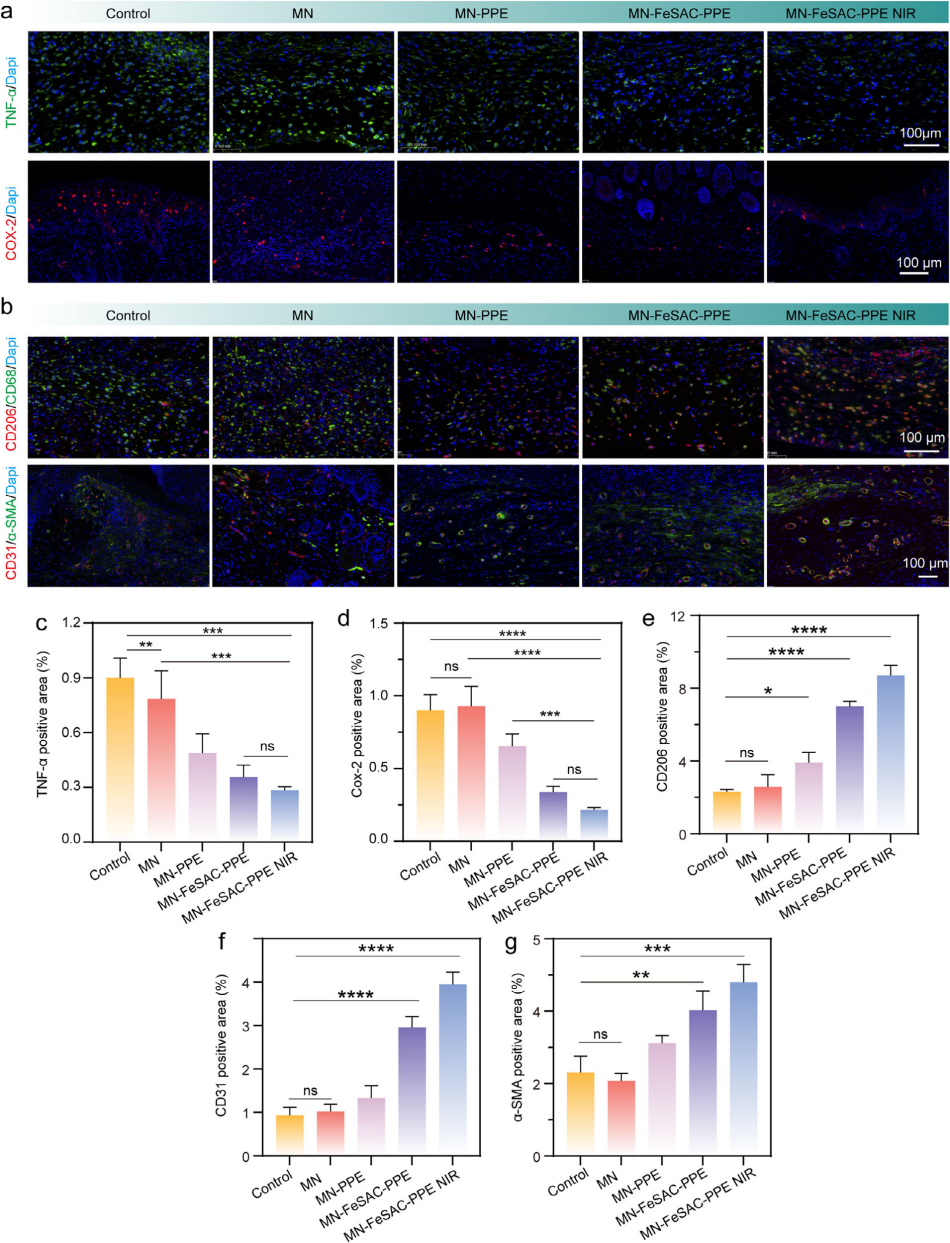
(a) Immunofluorescence staining images of TNF‐α (green) and COX‐2 (red). (b) Representative immunofluorescence staining of macrophage marker (CD206 and CD68) and vascularization marker (CD31 (red) and α‐SMA (green)) within the diabetic infected wound area. (c, d) Quantitative evaluation of fluorescence intensity for TNF‐α and COX‐2. (e) Quantitative evaluation of the positive area measurements for CD206. (f, g) Quantitative assessment of regions positive for CD31 and α‐SMA. All data are shown as the mean ± standard deviation (SD). n = 3, ^*^ *P* < 0.05, ^**^ *P* < 0.01, ^***^ *P* < 0.001.

## 3 Conclusion

We developed a stage‐adaptive Janus microneedle patch, MN‐FeSAC‐PPE, for infected diabetic wound repair by integrating Fe‐SACs‐mediated antibacterial therapy with PPE‐mediated redox‐immune and bioenergetic regulation. In the early infection stage, NIR activation of Fe‐SACs nanozymes enabled efficient photothermal and catalytic antibacterial effects, thereby reducing the bacterial burden in infected diabetic wounds. Subsequently, PPE delivered from the microneedle tips exerted antioxidant and anti‐inflammatory effects by scavenging excessive ROS, attenuating inflammatory activation, and protecting cells from oxidative stress‐induced mitochondrial dysfunction. In vitro studies demonstrated that MN‐FeSAC‐PPE suppressed pro‐inflammatory cytokine expression, enhanced antioxidant defense, preserved mitochondrial membrane potential, and restored ATP production under oxidative stress conditions. Transcriptomic analysis further supported these findings, showing that MN‐FeSAC‐PPE downregulated representative inflammation‐related genes, including Tnf, Il1b, and Il6, while enhancing mitochondrial energy metabolism‐associated signatures, such as oxidative phosphorylation and citrate cycle‐related gene sets. In vivo, NIR‐activated MN‐FeSAC‐PPE significantly accelerated the healing of *S. aureus*‐infected diabetic wounds by suppressing inflammation, promoting anti‐inflammatory macrophage polarization, enhancing angiogenesis, and facilitating collagen deposition and tissue remodeling. Overall, this study presents a promising stage‐adaptive microneedle platform for coordinating antibacterial intervention, redox‐immune remodeling, and mitochondrial metabolic restoration in infected diabetic wound healing.

## 4 Experimental Section

### 4.1 Reagents and Materials

g‐C_3_N_4_, dopamine hydrochloride, Fe(NO_3_)_3_•9H_2_O, methylene blue (MB), 3,3′,5,5′‐tetramethyl‐benzidine (TMB), DPPH, ABTS, PTIO were sourced from Aladdin Biochemical Technology Co., Ltd (Shanghai, China). Cell Counting Kit‐8, calcein‐AM/PI, ROS detection kit (DCF‐DA), 6’‐diamidino‐2‐phenylindole (DAPI), Actin‐Tracker Red‐555, Mito‐Tracker Red, Mitochondrial membrane potential assay kit with JC‐1 were supplied from Beyotime Biotechnology (Shanghai China). The Live/Dead Bacterial Staining Kit employed in the experiments was obtained from Yeasen Biotechnology Co., Ltd. (Shanghai, China). The γ‐PGA used in this study (Mw 1000–15 000) was supplied by Sai Taisi Biological Technology Co., Ltd. (Nanjing, China). Microneedle patch molds (10 × 10 array; individual tip dimensions 200 µm × 200 µm × 600 µm, W × L × H) were procured from Shiling Laike Die Business Co., Ltd. (Guangzhou, China). Cell culture reagents, including Dulbecco's Modified Eagle Medium (DMEM) and fetal bovine serum (FBS), were obtained from Gibco (UK). PPE was purchased from Xi'An TongZe Biotech Co., Ltd. The propolis content and total flavonoid content were 40.00% and 40.03%, respectively, according to the supplier's certificate of analysis.

### 4.2 Synthesis of Fe‐SACs

Porous g‐C_3_N_4_ nanosheets were prepared by pyrolysis and condensation of urea. Briefly, g‐C_3_N_4_ nanosheets (100 mg) were dispersed in 100 mL of 10 mm Tris buffer and sonicated for 30 min. Dopamine hydrochloride (100 mg) was then added, and the mixture was stirred at room temperature for 3 h to form PDA‐coated g‐C_3_N_4_ nanosheets. The resulting PDA@g‐C_3_N_4_ was collected, washed, and re‐dispersed in an aqueous Fe(NO_3_)_3_·9H_2_O solution, followed by stirring for 12 h to coordinate Fe^3^^+^ ions with the PDA layer. After filtration and washing to remove unbound Fe^3^^+^, the Fe^3^^+^‐PDA@g‐C_3_N_4_ precursor was dried and pyrolyzed at 1000°C for 1 h under a nitrogen atmosphere, yielding Fe‐SACs with atomically dispersed Fe sites embedded in a nitrogen‐doped carbon scaffold.

### 4.3 Characterization of Fe‐SACs

The morphology and particle size of the Fe‐SACs were examined using transmission electron microscopy (TEM, JEOL Ltd., Tokyo, Japan). Morphological imaging by aberration‐corrected STEM was performed on a JEOL JEM‐ARM200F transmission electron microscope fitted with a probe spherical‐aberration corrector and operated at 200 kV. X‐ray absorption spectroscopy was undertaken at the Australian Synchrotron (ANSTO) using its dedicated XAS beamline in Melbourne. Fe foil, Fe_2_O_3_ and FeO were used as references and measured simultaneously.

### 4.4 Peroxidase‐Like Catalytic Activity and Photothermal Performance of Fe‐SACs

•OH was detected via oxidation of the TMB probe, producing enhanced absorbance at 652 nm. TMB served as an indicator to visualize and quantify •OH generation. An acetate‐buffered solution (1.0 mL) comprising Fe‐SAC at varying concentrations (10, 30, 45, 80, 120, 150, 200 µg·mL^−^^1^), 0.5 mm TMB and H_2_O_2_ (0, 0.01, 0.15, 0.2, 1.0 mm) was incubated at 25°C for 30 min. Subsequent UV‐vis‐NIR spectral measurements were performed to quantify absorbance variations. The catalytic behavior of Fe‐SACs was examined using methylene blue as a probe: reaction mixtures (FeSAC 200 µg·mL^−^^1^, MB 10 µg·mL^−^^1^, H_2_O_2_ 1.0 mm) were incubated for 30 min, MB degradation was monitored at 664 nm, and ESR spectroscopy confirmed the formation of •OH and ^1^O_2_. The production of •OH was attributed to the Fenton reaction between Fe‐SAC_S_ and H_2_O_2_. Singlet oxygen was detected using TEMP as the spin trap, while hydroxyl radicals were captured with DMPO. The photothermal properties of Fe‐SACs were evaluated under 808 nm NIR irradiation. The temperature changes were recorded using an infrared thermal imager with experiments conducted at a power density of 1 W/cm^2^ for 5 min.

### 4.5 Fabrication of MN Patches

Microneedle patch mold (8 mm × 8 mm and containing a 10 × 10 microneedle array) was fabricated using an electro‐discharge machining process provided by Shiling Laike Die Business Co., Ltd. For the tip layer, 3 mg γ‐PGA was dissolved in 1 mL deionized water, followed by the addition of 3 mg PPE powder. After ultrasonication, the PPE‐γ‐PGA hydrogel was added into the mold and filled into the microneedle tips under vacuum‐assisted negative pressure. For the backing layer, 3 mg γ‐PGA was dissolved in 1 mL deionized water, followed by the addition of 3 mg Fe‐SACs powder. The Fe‐SACs‐γ‐PGA hydrogel was then added to fill the backing layer. Finally, the mold was dried at 40°C for 3 h, and the dried MN‐FeSAC‐PPE patch was peeled from the mold and stored in a vacuum desiccator until use.

### 4.6 Mechanical Characterization

The mechanical properties of the fabricated MN and MN‐FeSAC‐PPE microneedle patches were evaluated using a uniaxial mechanical tester (HY‐940FS, Heng Yu Instrument Co., Ltd., Shanghai, China).

### 4.7 Characterization of Drug Permeability

The transdermal drug permeability of the MN patches was investigated using agarose hydrogel and mouse skin as models to simulate human skin tissue. MN‐FeSAC‐PPE patches loaded with Rhodamine B, serving as a model drug, were applied to mouse skin in vivo for 5 min. The skin treated with the MN‐FeSAC‐PPE patches were subsequently examined using a stereoscopic microscope under both bright‐field and Cy5‐field imaging. After that, the mouse skin samples were sectioned into 5 µm‐thick slices using a cryostat (Leica RM2235) for subsequent hematoxylin and eosin (H&E) staining. To assess penetration, the MN patch labeled with Rhodamine B dye was applied to an agarose hydrogel substrate for 5 min. Ten minutes post‐application, a representative image of the hydrogel was captured using confocal microscopy. 3D reconstructions were utilized to visualize the diffusion and penetration of the Rhodamine B dye from the MN patch into the agarose hydrogel.

### 4.8 Live/Dead Viability In Vitro

In vitro viability of MN, MN‐PPE, and MN‐FeSAC‐PPE was tested with a transwell insert. NIH‐3T3 cells (2.5 × 10^4/well) were seeded in 24‐well plates, cultured for 24 h, and then treated with MN, MN‐PPE, or MN‐FeSAC‐PPE (808 nm NIR). Cell survival was evaluated using a calcein AM/PI staining kit (Beyotime, Jiangsu, China), and fluorescence images were captured with an Olympus IX2‐ILL30 microscope.

### 4.9 Hemolysis Assay

To evaluate hemocompatibility, a 4% erythrocyte suspension was incubated with MN, MN‐PPE, MN‐FeSAC‐PPE, or MN‐FeSAC‐PPE under 808 nm NIR irradiation at 37°C for 4 h. Deionized water was used as the positive control, while PBS containing purified RBCs served as the negative control. After incubation, samples were centrifuged at 1500 rpm for 10 min to separate the cells. Subsequently, 100 µL of the cell‐free supernatant was collected into a 96‐well plate, and the absorbance at 540 nm was recorded using a microplate reader to quantify hemoglobin release. The percentage of hemolysis was calculated using the formula: Hemolysis% = (Ab_test_ − Ab_neg_)/(Ab_pos_ − Ab_neg_) × 100%, with Ab_test_, Ab_pos_, and Ab_neg_ denoting absorbance values for the test, positive, and negative groups, respectively.

### 4.10 Scratch Wound‐Healing Assay

Cells were plated in 6‐well dishes at 2 × 10^5^ cells/well. Once confluent, a sterile pipette tip was used to generate a consistent scratch in the monolayer, after which PBS washing removed loose cells. The cells were subsequently treated with MN, MN‐PPE, MN‐FeSAC‐PPE, MN‐FeSAC‐PPE under NIR, or standard culture medium (control). Following 24 h incubation, migration was assessed via an inverted fluorescence microscope.

### 4.11 Transwell Migration Analysis

A suspension of NIH‐3T3 cells (1 × 10^4^/well, 100 µL DMEM + 1% FBS) was added to the upper chambers of 24‐well Transwells (Corning, USA), whose inserts contained MN, MN‐PPE, MN‐FeSAC‐PPE, MN‐FeSAC‐PPE + NIR, or medium only (control). The plates were subsequently maintained in a humidified incubator at 37°C with 5% CO_2_ for 24 h. Post‐incubation, unmigrated cells on the insert tops were swabbed away. Migrated cells were crystal violet–stained (Beyotime, China) and imaged using a Carl Zeiss microscope (Oberkochen, Germany).

### 4.12 Tube Formation Analysis

A 96‐well plate was coated with 50 µL growth factor‐reduced Matrigel (BD Biosciences, USA), polymerized at 37°C for 30 min. Next, HUVECs were seeded on top of the Matrigel surface at a density of 1.5 × 10^4^ cells per well. Cells were treated with MN, MN‐PPE, MN‐FeSAC‐PPE, MN‐FeSAC‐PPE plus NIR irradiation, or untreated (control). After 6 h at 37°C in 5% CO_2_, tube‐like structures were imaged using a Carl Zeiss inverted microscope (Oberkochen, Germany). Angiogenic parameters, including node count, junctions per mm^2^, and mean tube length, were quantified with ImageJ software (National Institutes of Health, USA).

### 4.13 In Vitro Antioxidant Tests

The radical‐neutralizing potential of the PPE solution was evaluated via several standard protocols. Antioxidant efficacy was specifically quantified through DPPH•, ABTS•^+^, PTIO•, and •OH radical scavenging tests. All measurements were conducted and analyzed using UV–vis spectrophotometry.

ABTS•^+^ was generated by mixing 0.2 mL of 7.4 mM ABTS with 0.2 mL of 2.6 mm potassium persulfate and incubating in the dark for 24 h, followed by 50‐fold dilution in PBS. PPE samples (0.6 mL) were reacted with 2.4 mL of diluted ABTS•^+^ for 10 min, and absorbance was recorded at 734 nm.

PTIO (0.05 mg/mL in PBS) was reacted with PPE samples (2 mL each, various dilutions) for 2 h at room temperature, and the decrease in PTIO radical absorbance at 557 nm was determined by UV–vis spectroscopy.

The capacity to neutralize •OH was determined as per the guidelines in the •OH scavenging kit (Nanjing Jiancheng Bioengineering Research Institute). Following the reaction, absorbance at 550 nm was assessed via UV–vis spectrophotometry to evaluate the leftover •OH radicals.

Radical Inhibition Calculation: Prior to UV–vis analysis, all specimens were spun at 10 000 rpm for 10 min to remove lingering PPE. Every determination was carried out in three replicates for data robustness. The scavenging efficiency (%) for each radical was computed with the equation below:

$$\mathrm{Inhibition}\;\left(\%\right)=\frac{A_{0}-A}{A_{O}}\times 100\%$$

where A_0_ indicates the absorbance of the blank (without PPE), and A represents the absorbance when PPE is present.

### 4.14 Protection Against Oxidative Damage by MN‐FeSAC‐PPE

The‐ Cells were then exposed to 100 µm H_2_O_2_ for 4 more hours to induce oxidative stress. The ability of the MN‐FeSAC‐PPE patch to counteract H_2_O_2_ and mitigate ROS was investigated. NIH‐3T3 cells were seeded into 24‐well plates and co‐incubated with the patch for 24 h. Cells were then exposed to media with 100 µm H_2_O_2_ for an extra 4 h. Intracellular ROS was detected using 10 µm DCFH‐DA probe, with subsequent visualization and analysis on a Leica microscope (Germany).

### 4.15 JC‐1 Assay for Mitochondrial Membrane Potential

Cells were set up for confocal imaging based on the specified experimental conditions. After that, they were washed with PBS, and 1 mL of growth medium was pipetted into each well. Then, 1 mL of JC‐1 staining reagent (Beyotime Biotech, China) was added per well and mixed evenly. Plates were held at 37°C for 30 min. Post‐incubation, the liquid was aspirated, and cells were rinsed twice using JC‐1 buffer. Hoechst stain was applied for 10 min. Following this, 2 mL of medium was introduced to each well. Images were captured on a ZEISS LSM900 confocal microscope (ZEISS, Germany).

### 4.16 Mitochondrial Morphology Assays

Cells were treated with 50 nm Mito‐Tracker Red CMXRos (Beyotime, Shanghai, China) for 30 min at 37°C and then washed three times with PBS. The cells were then counterstained with Hoechst for 10 min to highlight nuclei. Stained samples were observed and documented using a ZEISS LSM900 confocal microscope (ZEISS, Germany).

### 4.17 The Transmission Electron Microscopy Analysis of Mitochondrial Morphology

Cells were fixed in 2.5% glutaraldehyde (0.1 m sodium acetate buffer, pH 7.4), post‐fixed in 2% osmium tetroxide, dehydrated with graded ethanol and propylene oxide, embedded in Epon resin (Merck) and polymerized (60°C, 24 h); ultrathin sections were stained with uranyl acetate (0.5%, 30 min) and lead citrate (3%, 7 min) at 20°C. Mitochondrial morphology was examined using a Zeiss Libra 120 Plus transmission electron microscope (Carl Zeiss NTS GmbH).

### 4.18 ATP Test

The effect of MN, MN‐PPE, and MN‐FeSAC‐PPE on NIH‐3T3 cell metabolism was examined by measuring ATP using a commercial kit (Beyotime, Shanghai, China). Cells (2.5 × 10^4^/well) were plated in 24‐well plates and treated for 24 h. Subsequently, cells were exposed to 100 µm H_2_O_2_ in culture medium for 4 h to induce oxidative stress. ATP secretion was evaluated in all groups by chemiluminescent measurement.

### 4.19 Photothermal Properties of MN, MN‐PPE and MN‐FeSAC‐PPE

The photothermal performance of MN, MN‐PPE, and MN‐FeSAC‐PPE patches was assessed under 808 nm NIR laser irradiation, with temperature changes monitored in real‐time using an infrared thermal imaging camera. Tests were performed at power densities of 0.3, 0.5, 0.7, and 1 W/cm^2^. Five on‐off NIR cycles were used to test the thermal stability of the MN‐FeSAC‐PPE patch, and the photothermal conversion efficiency (η) was determined from thermal equilibrium.

$\eta =\frac{\;\mathrm{hS}\left(T_{\max}-T_{\mathrm{surr}}\right)-Q_{0}}{I}$ where h represents the heat transfer coefficient, S (cm^2^) corresponds to the scaffold surface area, and I (W cm^−^^2^) denotes the laser power density applied. T_max indicates the equilibrium scaffold temperature, while T_surr refers to the ambient temperature (fixed at 25°C). All temperature values are expressed in degrees Celsius.

### 4.20 In Vitro Antibacterial Assay

The antibacterial performance of the microneedle (MN) patches was assessed against Escherichia coli (ATCC 25922) and Staphylococcus aureus (ATCC 25923) using the spread‐plate assay. Groups included MN, MN‐PPE, MN‐FeSAC‐PPE, MN‐FeSAC‐PPE with NIR irradiation (808 nm, 0.7 W/cm^2^, 10 min), and untreated controls. Patches were immersed in bacterial suspensions (1 × 10^8^ CFU/mL) and incubated for 5 h. Suspensions were diluted to 1 × 10^4^ CFU/mL in PBS, and 100 µL was plated on LB agar, followed by incubation at 37°C for 16–18 h. Colonies were counted, and viability (%) was calculated as (N_t / N_c) × 100%, where N_t and N_c are colony counts for treatment and control groups, respectively. Bacterial morphology on patches was assessed by SEM: samples were fixed in glutaraldehyde, dehydrated in graded ethanol (30%–100%), air‐dried, gold‐sputtered, and imaged.

### 4.21 Live/Dead Bacterial Staining

Bacterial viability was assessed via the Live/Dead Bacterial Staining Kit (YEASEN, Shanghai, China), where live cells fluoresced green (DMAO) and dead cells red (EthD‐III). Treated bacterial suspension (1 mL) was aliquoted into a 1.5 mL tube, combined with 1 µL DMAO and 2 µL EthD‐III, and incubated in the dark for 15 min. Subsequently, 10 µL was mounted on a glass slide, and fluorescence images were captured using a Leica LCS‐SP8‐STED confocal microscope (Germany).

### 4.22 NPN Uptake Assays

N‐phenyl‐1‐naphthylamine (NPN) was used to assess bacterial membrane permeability. A 1 mM NPN stock was diluted to 40 µm in 30% DMSO as the working solution. *E. coli* and *S. aureus* were grown on a shaker for 24 h, and 10^8^ CFU/mL were added to 24‐well plates. Suspensions were treated with MN, MN‐PPE, MN‐FeSAC‐PPE, or MN‐FeSAC‐PPE plus NIR for photothermal effects. Post‐treatment, 150 µL aliquots were transferred to 96‐well plates and incubated with 50 µL of 40 µm NPN at 37°C for 30 min. Fluorescence was measured at 420 nm emission after 350 nm excitation.

### 4.23 qPCR Analysis

Total RNA was isolated from cells treated with PPE or MN‐FeSAC‐PPE using TRIzol reagent (Thermo Fisher, USA) following the manufacturer's guidelines. RNA integrity was verified by spectrophotometry, with samples exhibiting A260/A280 ratios close to 2.0 considered appropriate for PCR. To investigate antioxidative responses, the expression of Gpx1, Cat, and Sod2 was examined. One microgram of RNA was reverse‐transcribed into cDNA using the Perfect Real‐Time RT kit (Takara, Japan) in a 20 µL reaction on a T100 Thermal Cycler (Bio‐Rad, Singapore). qPCR was subsequently performed with TB Green Premix ExTaq II (Takara, Japan) on a CFX96 Optical Module (Bio‐Rad, Singapore). Relative expression levels were analyzed using the 2^^−ΔΔCt^ method, normalizing target gene expression to the control group based on Ct values.

### 4.24 Transcriptome Sequencing and Data Analysis

RAW264.7 macrophages, a murine macrophage‐like cell line derived from Abelson murine leukemia virus‐induced tumors in BALB/c mice, were cultured in DMEM supplemented with 10% fetal bovine serum and 1% penicillin‐streptomycin at 37°C in a humidified atmosphere containing 5% CO_2_. RNA sequencing was performed to compare the mRNA expression profiles of RAW 264.7 cells in the LPS and MN‐FeSAC‐PPE groups, aiming to investigate the effects of MN‐FeSAC‐PPE on inflammatory regulation and mitochondrial energy metabolism. Briefly, RAW 264.7 cells were treated under LPS‐induced inflammatory conditions and co‐cultured with MN‐FeSAC‐PPE for 24 h. Total RNA was extracted using TRIzol reagent and stored at −80°C before sequencing. RNA libraries were constructed using the Illumina TruSeq RNA Sample Preparation Kit and sequenced by Majorbio Biotech Co., Ltd. (Shanghai, China). The sequencing data were analyzed using the Majorbio Cloud Platform. Differentially expressed genes (DEGs) between the LPS and MN‐FeSAC‐PPE groups were identified, followed by PCA, volcano plot, clustering heatmap, KEGG pathway enrichment analysis, and GSEA. Pathways with a corrected *P* value < 0.05 were considered significantly enriched.

### 4.25 In Vivo Evaluation of Antibacterial Effect and Wound Healing

All animal experiments were reviewed and approved by the Animal Care and Use Committee of Shanghai Tenth People's Hospital, Tongji University School of Medicine (Approval ID: SHDSYY‐2024‐44850901). Briefly, 8‐week‐old C57BL/6 mice were used in this study. After arrival, the mice were acclimatized for one week under SPF housing conditions before model establishment. Diabetes was induced by intraperitoneal injection of freshly prepared streptozotocin (STZ) dissolved in sodium citrate buffer. STZ was administered at a dose of 50 mg/kg once daily for five consecutive days. After STZ administration, fasting/random blood glucose levels were monitored regularly. Mice with blood glucose levels ≥14 mmol/L that remained stable for several consecutive days were considered successfully diabetic. After confirmation of the diabetic status, an infected full‐thickness skin wound model was established. Mice were anesthetized with isoflurane, and bilateral 6‐mm full‐thickness dorsal skin wounds were created. Twenty‐five mice were randomized into five groups: untreated control, MN, MN‐PPE, MN‐FeSAC‐PPE, and MN‐FeSAC‐PPE with NIR (808 nm, 0.7 W/cm^2^). On day ‐1 post‐wounding, wounds were inoculated with *Staphylococcus aureus*. Treatments commenced on day 0, with mice housed individually and provided ad libitum food and water. Digital images of wounds were taken on days 0, 2, 3, 5, 7, 9, 12, and 14. Wound areas were quantified using Image‐Pro Plus software.

### 4.26 Histology and Immunohistochemistry Staining

After a 14‐day treatment period, mouse were euthanized, and wound tissues were collected. Samples were fixed in 4% paraformaldehyde for 24 h, embedded in paraffin, and sectioned into 5 µm slices for subsequent analysis. Granulation tissue and collagen deposition were evaluated via H&E and Masson's trichrome staining, respectively. Immunofluorescence staining assessed inflammatory markers (CD86, CD206, COX‐2, TNF‐α) and angiogenic proteins (CD31, α‐SMA). Capillary density was quantified using Image‐Pro Plus 6 software (Media Cybernetics, USA).

### 4.27 Statistical Analysis

All quantitative results are presented as mean±SD. Intergroup variations were analyzed by one‐way ANOVA, followed by Tukey's test for post‐hoc comparisons. Statistical significance was defined as ^*^ *p* < 0.05, ^**^ *p* < 0.01, ^***^ *p* < 0.001, and ^****^ *p* < 0.0001.

## Conflicts of Interest

The authors declare no conflict of interest.

## Supporting information




**Supporting File**: advs76397‐sup‐0001‐SuppMat.docx.

## Data Availability

The data that support the findings of this study are available from the corresponding author upon reasonable request.
