# Supplementary material for: Stage‐Adaptive Janus Microneedle System for Redox‐Immune Regulation and Mitochondrial Protection in Infected Diabetic Wound Healing
Source: Adv Sci (Weinh). 2026 Jul 29:e00076. Online ahead of print. doi: 10.1002/advs.202600076 (PMC13418274; doi:10.1002/advs.202600076)
Supplement: Supplementary file 1 — Supporting File: advs76397‐sup‐0001‐SuppMat.docx. [file ADVS-9999-e00076-s001.docx]

**Stage-Adaptive Janus Microneedle System for Redox-Immune Regulation and Mitochondrial Protection in Infected Diabetic Wound Healing**

*Mengting Yin^1,a,b^, Yu Zhang ^1,c^, Xinyu Qu^1,b^,Jiayi Liu^d^, Zhongyi Sun^,b^, Haibo Liu ^a^, Ziyan Chen ^b^, Jing Ru ^b^,* *Jingwen Han^a^, Bingqiang Lu ^a^,* *Yan Lu^c^, Yan Wang^*,a^, Xinyu Zhao^*,b^ and* *Feng Chen^*,a,b^*

^a^Shanghai Key Laboratory of Craniomaxillofacial Development and Diseases, Shanghai Stomatological Hospital & School of Stomatology, Fudan University, Shanghai 201102, P.R. China.

^b^Center for Orthopaedic Science and Translational Medicine, Department of Orthopaedics, Shanghai Tenth People’s Hospital, School of Medicine, Tongji University Shanghai 200072, P. R China.

^c^ State Key Laboratory of High Performance Ceramics and Superfine Microstructure, Shanghai Institute of Ceramics, Chinese Academy of Science, Shanghai 200050 P. R. China.

^d^Shenghua Zizhu Academy, shanghai 200241 P. R. China.

^1^Authors have equally contributed to this work: Mengting Yin, Yu Zhang, Xinyu Qu.

E-mail addresses:

[xyzhao@tongji.edu.cn,](mailto:xyzhao@tongji.edu.cn,) kq_wangyan@fudan.edu.cn, chen_feng@fudan.edu.cn


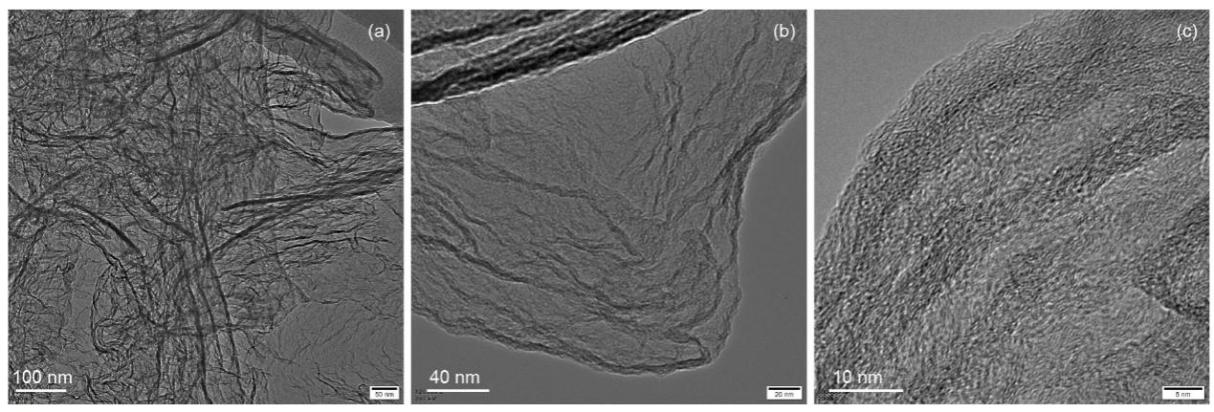


Figure S1. TEM images of synthetic Fe-SACs. (a) Scale bar of 100 nm. (b) Scale bar of 40 nm. (c) Scale bar of 10 nm.


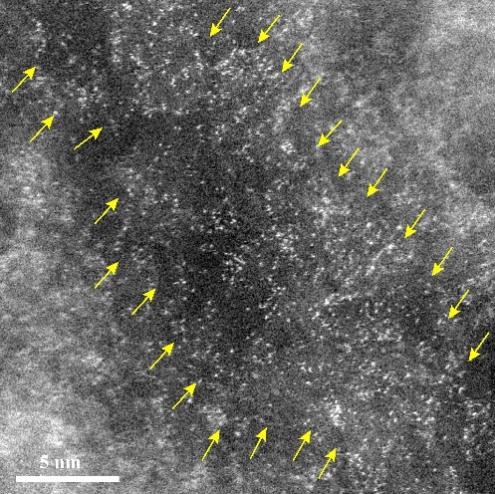


Figure S2. AC-STEM image of Fe-SACs.


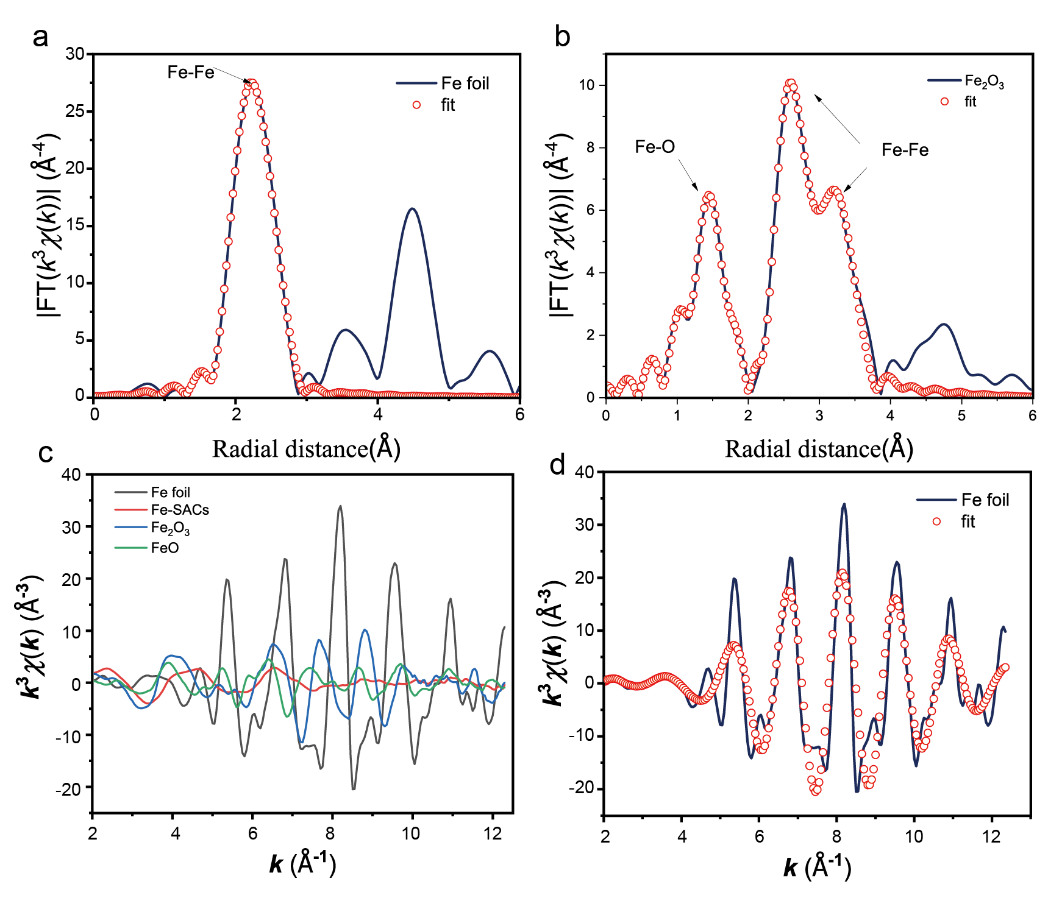


Figure S3. Fourier transformed Fe *k*-edge EXAFS spectra of Fe-SACs, Fe_2_O_3_, and Fe foil in R-space.

| Sample | Shell | *CN^a^* | *R*(Å)*^b^* | *σ*^2^(Å^2^)*^c^* | Δ*E*_0_(eV)*^d^* | *R* factor |
| --- | --- | --- | --- | --- | --- | --- |
| Fe foil | Fe-Fe | 8* | 2.467±0.014 | 0.0049±0.0014 | 5.9±2.2 | 0.0024 |
|  | Fe-Fe | 6* | 2.846±0.010 | 0.0054±0.0025 |  |  |
| Fe_2_O_3_ | Fe-O | 6.1±0.2 | 1.953±0.026 | 0.0134±0.0036 | -6.1±4.4 | 0.0107 |
|  | Fe-Fe | 3.9±0.8 | 2.963±0.014 | 0.0063±0.0015 | 1.8±2.2 |  |
|  | Fe-Fe | 2.9±0.8 | 3.389±0.019 |  |  |  |
|  | Fe-Fe | 3.9±1.2 | 3.697±0.015 |  |  |  |
| Fe-SACs | Fe-N | 4.4±0.4 | 1.939±0.010 | 0.0139±0.0017 | 2.5±1.2 | 0.0023 |
|  | Fe-N-C | 1.3±0.7 | 2.937±0.031 | 0.0051±0.0004 | 9.9±3.5 |  |

Table S1. EXAFS fitting parameters at the Fe K-edge for various samples（Ѕ02=0.785）*^a^CN*, coordination number; *^b^R*, the distance to the neighboring atom; *^c^σ*^2^, the Mean Square Relative Displacement (MSRD); *^d^ΔE*_0_, inner potential correction; *R* factor indicates the goodness of the fit. *S*0^2^ was fixed to 0.785, according to the experimental EXAFS fit of Fe foil by fixing *CN* as the known crystallographic value. * This value was fixed during EXAFS fitting, based on the known structure of Fe. Fitting range: 3.0 ≤ *k* (/Å) ≤ 11.6 and 1.0 ≤ *R* (Å) ≤ 3.0 (Fe foil); 3.0 ≤ *k* (/Å) ≤ 12.4 and 1.0 ≤ *R* (Å) ≤ 4.0 (Fe2O3); 2.0 ≤ *k* (/Å) ≤ 10.0 and 1.0 ≤ *R* (Å) ≤ 3.0 (YMT_Fe1). A reasonable range of EXAFS fitting parameters: 0.700 < *Ѕ*_0_^2^ < 1.000; *CN >* 0; *σ*^2^ > 0 Å^2^; |Δ*E*_0_| < 10 eV; *R* factor < 0.02.


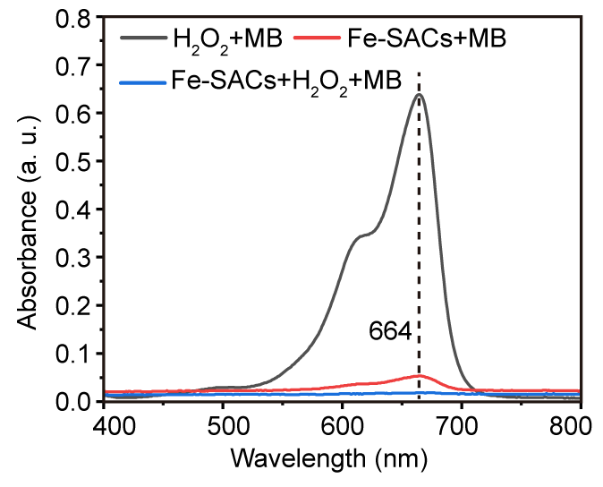


Figure S4. UV–vis absorption spectra of MB + H_2_O_2_, MB + FeSACs, and MB + FeSACs + H_2_O_2_.


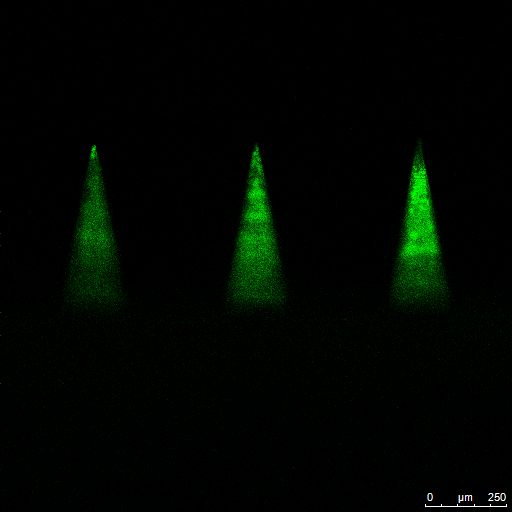


Figure S5. Fluorescence images of PPE@FITC loaded into the microneedle tips.


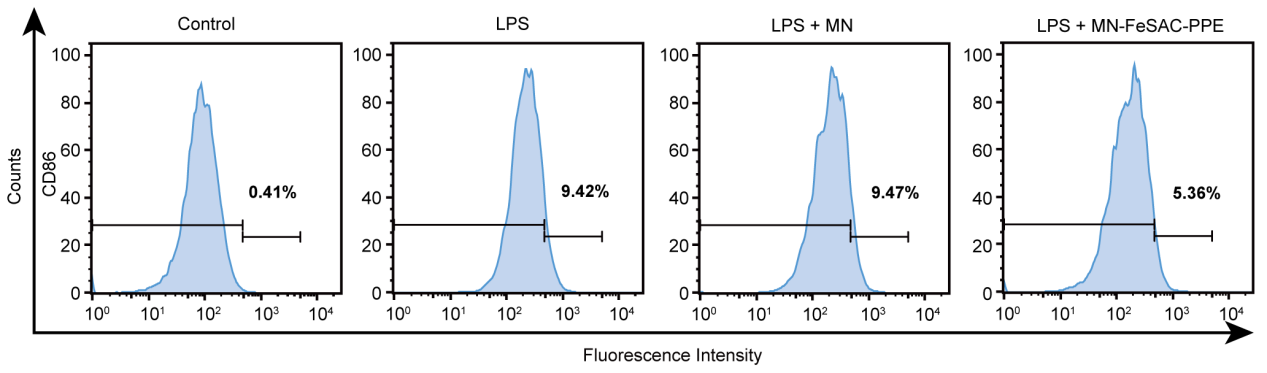


Figure S6. Flow cytometry results showing CD86 expression of macrophages with different treatments and represented as flow histograms.


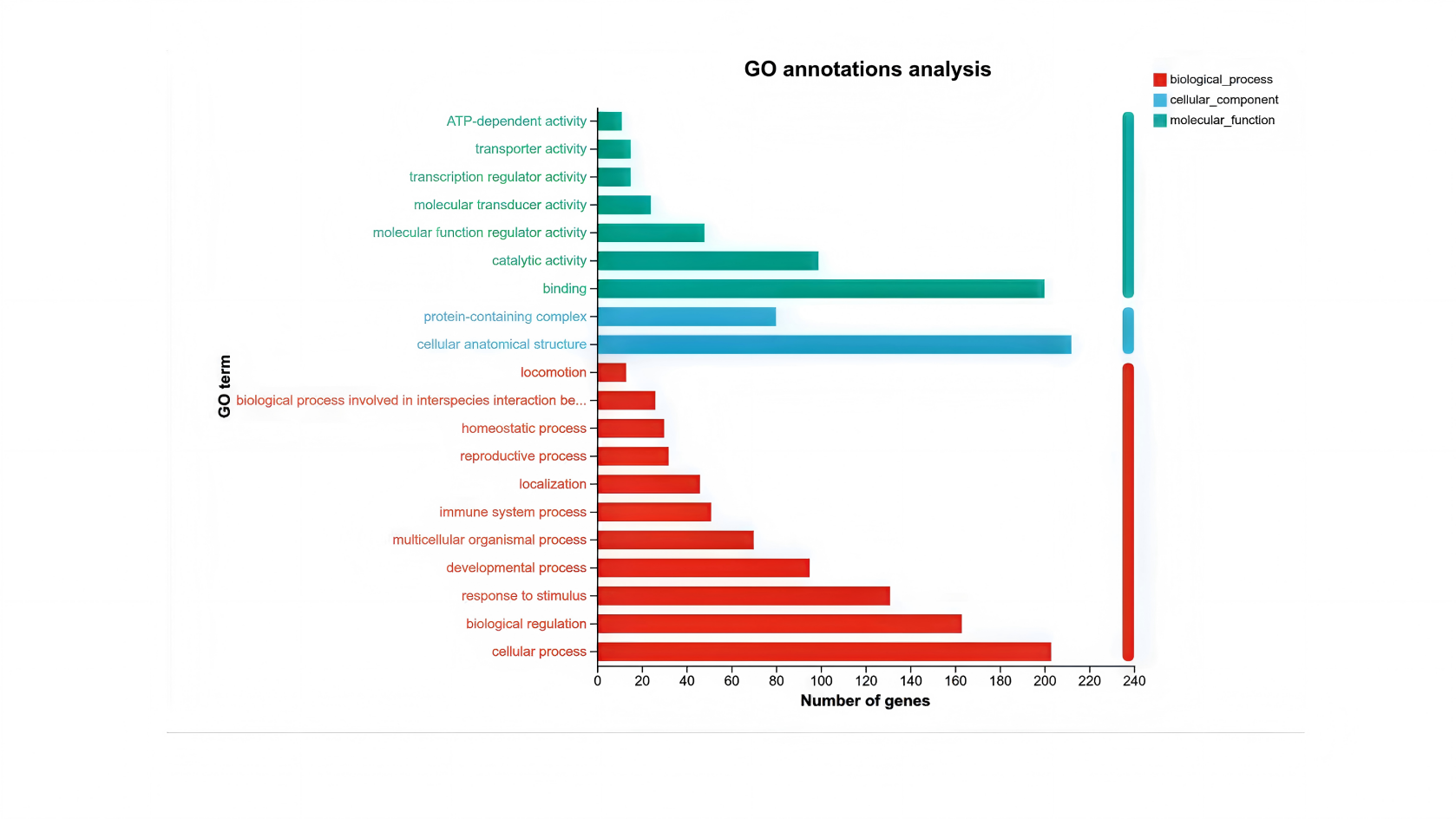


Figure S7. GO enrichment analysis showing the distribution of differentially expressed genes across biological process, cellular component, and molecular function categories.


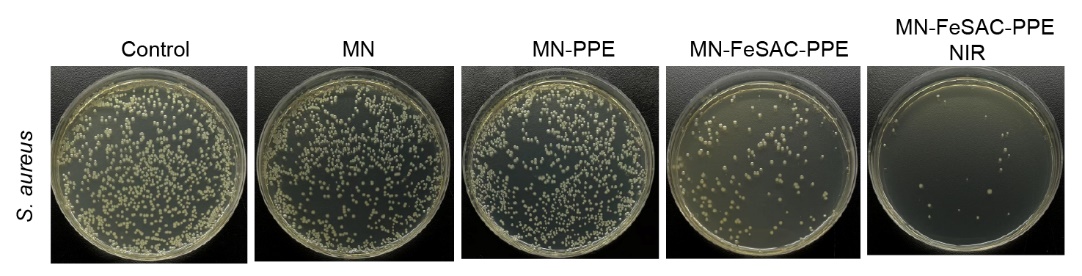


Figure S8. Representative images depicting spatial distribution of bacterial colonies on wound surfaces at Day 3 post-treatment across different groups.


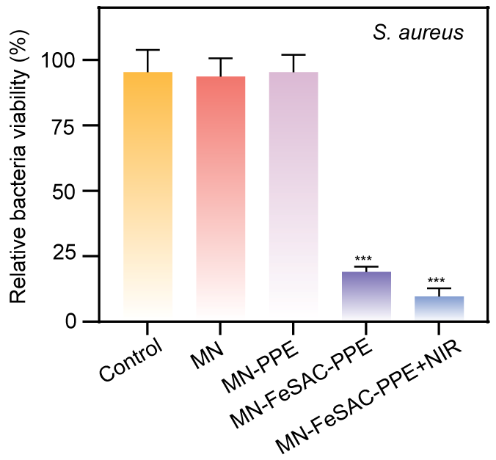


Figure S9. The relative viability of bacteria treated with different groups.


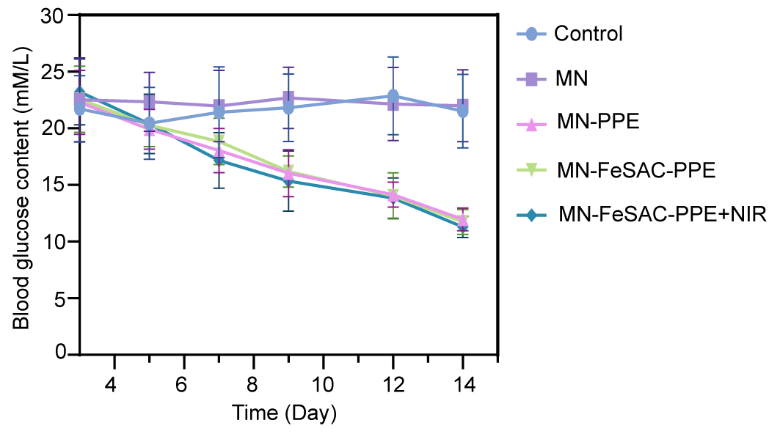


Figure S10. Blood glucose levels in mice among different treatment groups at different time points.


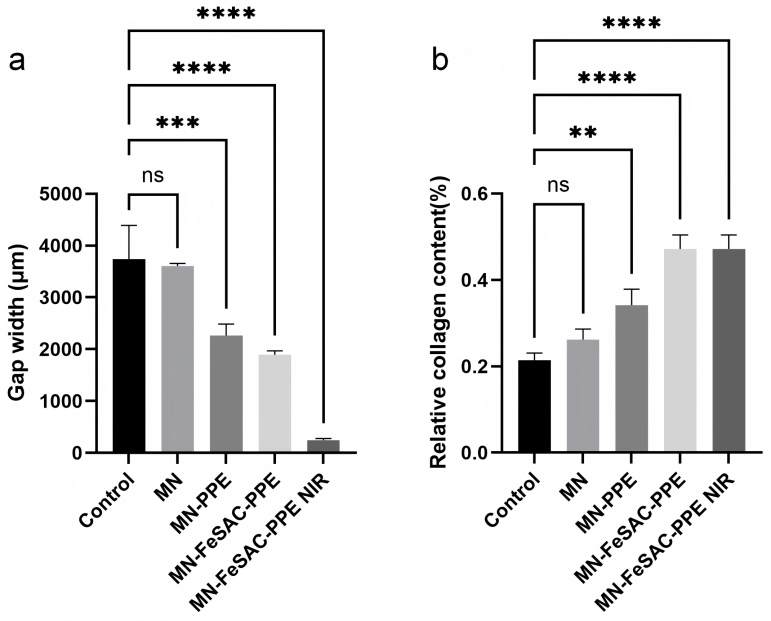


Figure S11. (a) The wound gap width of the different treatment groups；（b）The relative collagen content of the different treatment groups.


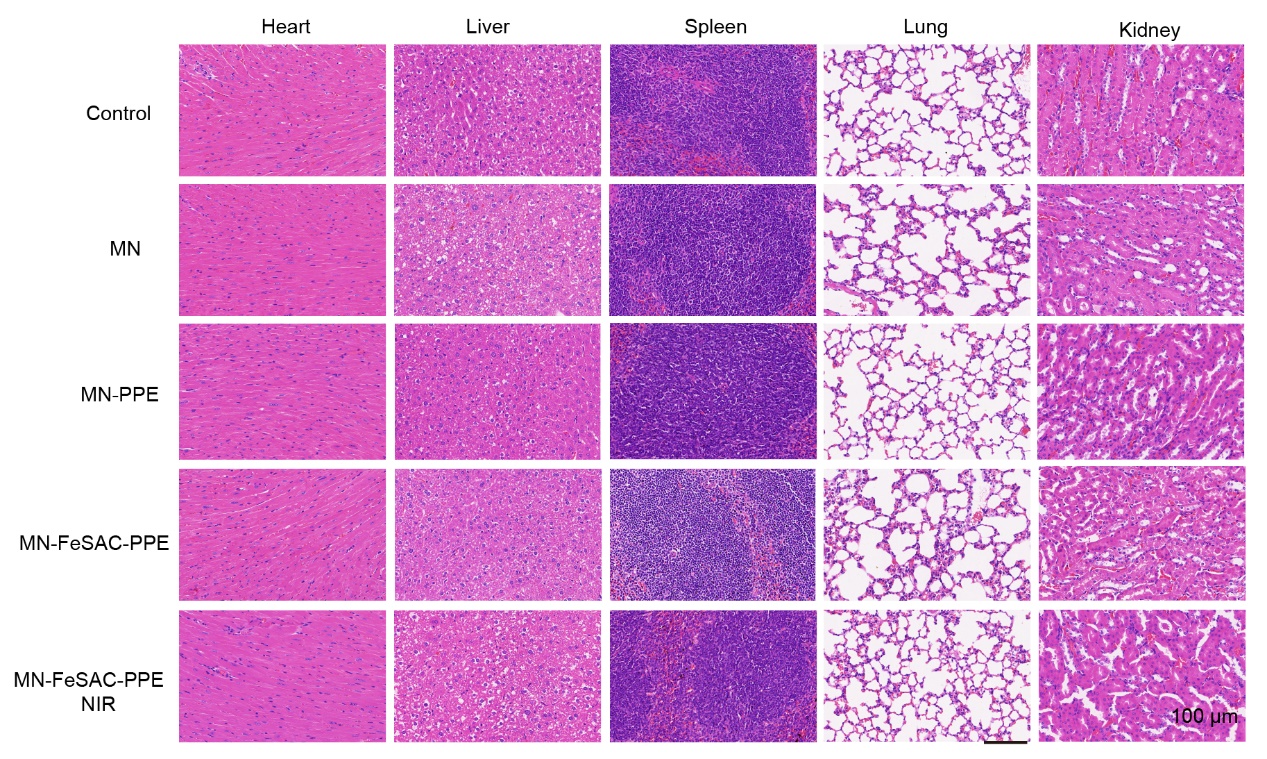


Figure S12. Histological analysis of major organs (Heart, Liver, Spleen, Lung, Kidney) by hematoxylin and eosin (H&E) staining. Scale:100 μm.
